# Comparison of univariate and multivariate analyses for brain [18F]FDG PET data in α-synucleinopathies

**DOI:** 10.1016/j.nicl.2023.103475

**Published:** 2023-07-13

**Authors:** Giulia Carli, Sanne K. Meles, Fransje E. Reesink, Bauke M. de Jong, Andrea Pilotto, Alessandro Padovani, Andrea Galbiati, Luigi Ferini-Strambi, Klaus L. Leenders, Daniela Perani

**Affiliations:** a Department of Nuclear Medicine and Molecular Imaging, University of Groningen, University Medical Center Groningen, Groningen, The Netherlands; b Department of Neurology, University of Groningen, University Medical Center Groningen, Groningen, The Netherlands; c School of Psychology, Vita-Salute San Raffaele University, Milan, Italy; d Department of Clinical Neuroscience, Sleep Disorders Center, San Raffaele Hospital, Milan, Italy; e Neurology Unit, Department of Clinical and Experimental Sciences, University of Brescia, Brescia, Italy; f In Vivo Human Molecular and Structural Neuroimaging Unit, Division of Neuroscience, IRCCS San Raffaele Scientific Institute, Milan; g Nuclear Medicine Unit, San Raffaele Hospital, Milan, Italy

**Keywords:** [18F]FDG-PET, Univariate analyses, SSM/PCA, Imaging biomarkers, α-synucleinopathies

## Abstract

**Background:**

Brain imaging with [18F]FDG-PET can support the diagnostic work-up of patients with α-synucleinopathies. Validated data analysis approaches are necessary to evaluate disease-specific brain metabolism patterns in neurodegenerative disorders. This study compared the univariate Statistical Parametric Mapping (SPM) single-subject procedure and the multivariate Scaled Subprofile Model/Principal Component Analysis (SSM/PCA) in a cohort of patients with α-synucleinopathies.

**Methods:**

We included [18F]FDG-PET scans of 122 subjects within the α-synucleinopathy spectrum: Parkinson’s Disease (PD) normal cognition on long-term follow-up (PD – low risk to dementia (LDR); n = 28), PD who developed dementia on clinical follow-up (PD – high risk of dementia (HDR); n = 16), Dementia with Lewy Bodies (DLB; n = 67), and Multiple System Atrophy (MSA; n = 11). We also included [18F]FDG-PET scans of isolated REM sleep behaviour disorder (iRBD; n = 51) subjects with a high risk of developing a manifest α-synucleinopathy. Each [18F]FDG-PET scan was compared with 112 healthy controls using SPM procedures. In the SSM/PCA approach, we computed the individual scores of previously identified patterns for PD, DLB, and MSA: PD-related patterns (PDRP), DLBRP, and MSARP. We used ROC curves to compare the diagnostic performances of SPM t-maps (visual rating) and SSM/PCA individual pattern scores in identifying each clinical condition across the spectrum. Specifically, we used the clinical diagnoses (“gold standard”) as our reference in ROC curves to evaluate the accuracy of the two methods. Experts in movement disorders and dementia made all the diagnoses according to the current clinical criteria of each disease (PD, DLB and MSA).

**Results:**

The visual rating of SPM t-maps showed higher performance (AUC: 0.995, specificity: 0.989, sensitivity 1.000) than PDRP z-scores (AUC: 0.818, specificity: 0.734, sensitivity 1.000) in differentiating PD-LDR from other α-synucleinopathies (PD-HDR, DLB and MSA). This result was mainly driven by the ability of SPM t-maps to reveal the limited or absent brain hypometabolism characteristics of PD-LDR. Both SPM t-maps visual rating and SSM/PCA z-scores showed high performance in identifying DLB (DLBRP = AUC: 0.909, specificity: 0.873, sensitivity 0.866; SPM t-maps = AUC: 0.892, specificity: 0.872, sensitivity 0.910) and MSA (MSARP: AUC: 0.921, specificity: 0.811, sensitivity 1.000; SPM t-maps: AUC: 1.000, specificity: 1.000, sensitivity 1.000) from other α-synucleinopathies. PD-HDR and DLB were comparable for the brain hypo and hypermetabolism patterns, thus not allowing differentiation by SPM t-maps or SSM/PCA. Of note, we found a gradual increase of PDRP and DLBRP expression in the continuum from iRBD to PD-HDR and DLB, where the DLB patients had the highest scores. SSM/PCA could differentiate iRBD from DLB, reflecting specifically the differences in disease staging and severity (AUC: 0.938, specificity: 0.821, sensitivity 0.941).

**Conclusions:**

SPM-single subject maps and SSM/PCA are both valid methods in supporting diagnosis within the α-synucleinopathy spectrum, with different strengths and pitfalls. The former reveals dysfunctional brain topographies at the individual level with high accuracy for all the specific subtype patterns, and particularly also the normal maps; the latter provides a reliable quantification, independent from the rater experience, particularly in tracking the disease severity and staging. Thus, our findings suggest that differences in data analysis approaches exist and should be considered in clinical settings. However, combining both methods might offer the best diagnostic performance.

## Highlights

• Substantial agreement between the SPM t-maps and the SSM/PCA pattern scores.
• The SPM single-subject maps had the best performance in identifying PD-LDR.
• SSM/PCA pattern scores reflect the disease staging and severity.

## 1 Introduction

Parkinson's Disease (PD), PD with dementia (PDD), Dementia with Lewy Bodies (DLB) and Multiple System Atrophy (MSA) compose the neuropathological spectrum of α-synucleinopathies (Goedert et al., 2013). PD is the most common form of α-synucleinopathy and is diagnosed based on the cardinal motor features (bradykinesia and rigidity and/or tremor) (Postuma et al. 2015). It is increasingly recognised that PD is a heterogeneous disorder that manifests a wide range of non-motor symptoms, including cognitive decline (Poewe et al., 2017). Cognitive impairment occurs in PD patients with a prevalence of 20% after five years of disease duration (Aarsland and Kurz, 2010), reaching 83% after ten years (Hely et al., 2008). After about 20 years of disease, cognitive deterioration is almost inevitable (Hely et al., 2008). The degree of cognitive decline varies, and subtypes exist (Kehagia et al., 2012). PDD is the clinical evolution of PD; however, it shares many clinical, neurochemical, and morphological features with DLB (Caminiti and Carli, 2023, Friedman, 2018). If cognitive impairment arises before the onset of the typical motor features, patients are diagnosed with DLB (McKeith et al., 2017). When a severe cognitive impairment arises after the onset of motor features, patients are diagnosed with PDD. This distinction (the so-called 1-year rule) might be arbitrary, and some consider PDD and DLB the same condition (Jellinger and Korczyn, 2018). On the other hand, despite sharing similarities, DLB and PDD might not necessarily behave the same way regarding the cognitive profiles (Smirnov et al., 2020). Thus, whether the differences between DLB and PDD are substantial enough not to be combined in interventional clinical trials it is still matter of debate (Armstrong and Emre, 2020).

MSA is the rarest of the three major α-synucleinopathies (Fanciulli and Wenning, 2015). In one study, only 62% of patients clinically diagnosed with MSA by community neurologists also had a diagnosis of MSA based on neuropathological features following the autopsy (Koga et al., 2015, Rizzo et al., 2016). The most common diseases clinically misdiagnosed as MSA include PD and DLB (Koga et al., 2015). This phenomenon is explained by profound autonomic failure, which can also occur in DLB (Kanazawa et al., 2007) and PD (Chaudhuri and Schapira, 2009). Due to the overlapping clinical features, it is sometimes challenging to make an early, accurate diagnosis of α-synucleinopathies (Brajkovic et al., 2017, Saeed et al., 2017). This hampers the proper treatment and prognostic counselling (Brajkovic et al., 2017).

Early, accurate diagnosis is pertinent for including patients in potential clinical trials. Therapeutic interventions for α-synucleinopathies may be more successful if prompted in the earliest disease phases. Isolated REM sleep behaviour disorder (iRBD) represents the prodromal stage of the α-synucleinopathies spectrum (Högl et al., 2018). *Postmortem* data, together with *in vivo* detection of phosphorylated α-synuclein deposits (i.e., biopsies of the colon, submandibular gland, and skin), demonstrated the presence of α-synuclein-related pathology in iRBD patients (Doppler et al., 2021, Fernández-Arcos et al., 2018, Iranzo et al., 2013). Of note, over a long-term follow-up, the estimated risk for iRBD patients to develop a full-blown α-synucleinopathy exceeds 90% (Galbiati et al., 2019). This usually includes PD (20–50% of patients) and DLB (20–40%); only a small percentage of iRBD patients (5–10%) develop MSA.

Biomarkers are needed to support clinical diagnosis. Brain imaging with [18F]FDG-PET appears promising because disease-specific brain hypo- and hypermetabolism patterns can be identified in parkinsonian syndromes (Perani et al., 2020; Teune et al., 2013). These patterns can be observed by a visual reading of a [18F]FDG-PET brain scan and represent a supportive marker in diagnostic criteria for DLB and MSA (Gilman et al., 2008; McKeith et al., 2017). In literature, two validated approaches can evaluate disease-specific brain metabolism patterns in α-synucleinopathies: the univariate Statistical Parametric Mapping (SPM) single-subject procedure (Della Rosa et al., 2014, Perani et al., 2014) and the multivariate Scaled Subprofile Model/Principal Component Analysis (SSM/PCA) (Eidelberg, 2009, Spetsieris et al., 2013). Both have limitations and a head-to-head comparison of these two methods has not yet been performed.

The univariate SPM single-subject procedure can identify specific hypometabolism patterns at the single-subject level, pertaining to the known prototypical patterns in each disease able to classify the subject (Perani et al., 2020). This semi-quantitative approach has high sensitivity and specificity in distinguishing atypical forms of parkinsonism (Caminiti et al., 2017) and predicting dementia development in PD patients (Pilotto et al., 2018). In DLB, this procedure has provided a ∼50% increase in accuracy compared with the first clinical assessment alone and an accuracy of >90% in distinguishing DLB from Alzheimer's dementia (AD) and PD (Caminiti et al., 2019). Notably, specific hypometabolism patterns can also be identified in iRBD patients indicating sensitivity in the earliest disease stages of the α-synucleinopathies spectrum (Carli et al., 2020).

SSM/PCA is a fully quantitative, data-driven method where disease-specific patterns are first determined at the group level in an identification cohort of healthy controls and patients. Then, the expression of the identified pattern can be prospectively quantified at the single-subject level (Spetsieris et al., 2013). The PD-related pattern (PDRP) (Schindlbeck and Eidelberg, 2018), MSA-related pattern (MSARP) (Tomše et al., 2022), and DLB-related pattern (DLBRP) (Perovnik et al., 2022)have been identified and validated. The SSM/PCA approach has been extensively applied in parkinsonian syndromes (Eckert et al., 2008, Ge et al., 2018, Ma et al., 2007, Meles et al., 2020, Niethammer et al., 2014) and also in iRBD (Kim et al., 2021, Kogan et al., 2020, Meles et al., 2017, Wu et al., 2014). Notably, *postmortem* confirmations further corroborated the validity of this method in idiopathic PD and MSA (Tang et al., 2010). SSM/PCA can accurately differentiate PD from atypical parkinsonism (Schindlbeck and Eidelberg, 2018), and MSA from non-MSA (AUC of 0.95) (Rus et al., 2020, Tang et al., 2010; Tripathi et al., 2016).

Given these promising results, a comparison between uni- and multivariate analysis is necessary. So far, this comparison has only been evaluated in AD (Habeck et al., 2008). That study demonstrated that both methods accurately differentiate between AD and healthy controls; however, the SSM/PCA approach benefits from better sensitivity, and the univariate approach occasionally suffered from false negatives. In this study, we evaluated the diagnostic accuracy of both methods in patients within the α-synucleinopathy spectrum. [18]FDG-PET images of 173 patients were retrospectively collected and analysed using both the univariate SPM single-subject and multivariate SSM/PCA methods.

## 2 Materials and methods

### 2.1 Participants

Isolated REM Sleep Behaviour Disorder (iRBD) – Fifty-one subjects with a polysomnography-confirmed diagnosis of iRBD (42 men, 9 women; age, 69±7 years) (American Academy of Sleep Medicine, 2014) were included in the study from the clinical database of the Sleep disorders Centre of Turro San Raffaele Hospital, Milan, Italy. The iRBD patients did not have dementia or motor symptoms at the time of diagnosis and imaging. Some of these patients (37/51) were described in a previous study (Carli et al., 2020). These subjects are part of a currently ongoing prospective project, and they all underwent motor (including Unified Parkinson's Disease Rating Scale part III (UPDRS-III)) and autonomic assessments, global cognitive efficiency testing (i.e. Mini-Mental State Examination (MMSE)), a standardised neuropsychological battery (Table 1). Details are described elsewhere (Carli et al., 2020).

**Table 1:** Demographical, clinical, and PET acquisition features.

	HC (N = 9)	iRBD (N = 51)	PD-LDR (N = 28)	PD-HDR (N = 16)	DLB (N = 67)	MSA (N = 11)	p-values
Gender (M/F)	4/5	42/9‡	16/12	11/5	34/33	6/5	0.000‡
Age, years	49.56±13.63	68.67±6.73*	62.68±10.83*	69.25±6.67	72.82±8.05*	66.56±7.26	0.000
Disease duration, years	–	6.25±3.16 ^a*^	4.25±2.59*	6.75±4.34*	2.40±1.94*	4.5±1.73^a^	0.000
UPDRS-III	–	1.55±2.48*	15.04±7.02*	16.63±7.58*	- b	- b	0.000
MMSE	–	28.00±2.74*	28.71±1.49	27.38±7.58*	18.41±4.92*	28.33±1.53 a	0.000
PET scanner	Discovery ST-E GE	Discovery ST-E GE	GE Discovery 3D PET/CT 690	GE Discovery 3D PET/CT 690	Discovery ST-E GE	Discovery ST-E GE	–
Reconstruction algorithm	OSEM	OSEM	OSEM	OSEM	OSEM	OSEM	–
Center	HSR-Milan	HSR-Milan	University of Brescia	University of Brescia	HSR-Milan	HSR-Milan	–

HC: Healthy Controls; iRBD: Isolated REM sleep Behaviour disorder; PD: Parkinson's disease; DLB: Dementia with Lewy Bodies; PD-LDR: PD with low risk of dementia; PD-HDR: PD with a high risk of dementia; MSA: Multiple System Atrophy; UPDRS-III: Unified Parkinson's Disease Rating Scale part III (motor); MMSE: Mini-Mental State Examination; PET: Positron Emission Tomography; OSEM: ordered subset expectation maximisation.

‡ iRBD patients showed different gender distribution compared to HC (p = 0.012, chi-square: 6.15), PDNC (p = 0.015, chi-square: 5.88), DLB (p = 0.000, chi-square: 12.62) and MSA (p = 0.045, chi-square: 4.00),

a Due to the retrospective nature of the studies, the exact disease duration is known for 4 MSA patients and 24 RBD patients. MMSE scores were available for only 3 MSA patients.

b The presence of motor symptoms in DLB and MSA was based on the neurological examination performed by experts in movement disorders. Specifically, in 35 out of 67 DLB patients presence of parkinsonism was reported.

* Denotes a significant difference (post-hoc comparison p < 0.05, Bonferroni corrected). Age: iRBD and PD stable were significantly younger than DLB; Disease Duration: DLB showed a lower disease duration than iRBD and PD stable; UPDRS-III: iRBD showed significantly lower scores than PD-LDR and PD-HDR; MMSE: DLB showed significantly lower MMSE scores than iRBD, PD-LDR and PD-HDR.

Parkinson's Disease (PD) – Forty-four patients with a clinical diagnosis of PD (Postuma et al. 2015) were included in the study from the clinical and imaging database of the Neurology Unit, Department of Clinical and Experimental Sciences, at the University of Brescia, Brescia, Italy. [18F]FDG-PET was performed at baseline when none of the patients had a diagnosis of dementia. After a 8-year (mean 8.9 ± 1.2 years, range: 8–10 years) clinical follow-up from [18F]FDG-PET imaging, 16 subjects developed dementia (11 men, 5 women; age, 69±7 years age in years), and 28 remained cognitively stable (16 men, 12 women; age, 63±11 years). For this reason, we refer to PD who developed dementia (N = 16) as PD at high risk of dementia (PD-HDR) and PD who did not (N = 28) as PD at low risk of dementia (PD-LDR).

Each patient underwent a standardised neurologic examination, including motor assessment, UPDRS-III, global cognitive efficiency (i.e., MMSE) and neurophysiological assessments. These patients are part of previously published data; details are described elsewhere (Pilotto et al., 2018).

Dementia with Lewy Bodies (DLB) – We included 67 patients with a clinical diagnosis of probable DLB (McKeith et al., 2017) from the clinical and imaging database of the Departments of Neurology and Nuclear Medicine Unit of San Raffaele Hospital (Milan, Italy) (34 men, 33 women; age, 73±8 years). The clinical assessments included medical history, and physical, neurological, MMSE and cognitive examination.

Multiple System Atrophy (MSA) – We retrospectively collected 11 patients with a diagnosis of probable MSA (Gilman et al., 2008), including 9 with dominant ataxia (MSA-C), and 2 with dominant parkinsonism (MSA-P) from the clinical and imaging database of the Departments of Neurology and Nuclear Medicine Unit of San Raffaele Hospital (Milan, Italy) (6 men, 5 women; age, 67±7 years age in years). The clinical assessments included medical history and physical and neurological assessments performed by neurologists with expertise in movement disorders.

Healthy controls (HC) – A group of 112 HC (53 men, 59 women; age, 65±9 years age in years) were included from the internal database of the In Vivo Human Molecular and Structural Neuroimaging Unit, IRCCS San Raffaele Scientific Institute, Milan, Italy. They presented a negative medical history of neurological or psychiatric diseases or other chronic illnesses and were not taking psychoactive medication. This database of normal controls has been implemented in the SPM single-subject procedure (see (Della Rosa et al., 2014, Perani et al., 2014)) and validated in different clinical contexts. This is the internal HC database of San Raffaele Hospital (HSR) Nuclear Medicine Unit, which is largely used for research purposes and in support of clinical routine. Caminiti and colleagues (2021) compared these HC with two other HC groups characterised by the absence of global cognitive impairment (as assessed by an MMSE score ≥ 28) and cognitive normality after an average 4-year clinical follow-up (for details on HC groups see (Caminiti et al., 2021)). The authors demonstrated that all the HC cohorts produced comparable maps of hypometabolism, thus, representing correct control cohorts for use in research and clinical settings.

The local ethical committees approved this study in conformity with the Helsinki Declaration. Informed consent was obtained from all participants or their informed caregivers.

### 2.2 [18F]FDG-PET image acquisition and reconstruction

All patients underwent [18F]FDG-PET scans performed with PET cameras manufactured by GE Healthcare medical systems (GE Healthcare, Chicago, IL): GE Discovery PET/CT 690 (PD cohort: PD-LDR and PD-HDR, 256×256 matrix with a voxel size of 1.95 mm) and STE scanner (DLB, MSA and iRBD, 128×128 matrix with a pixel size of 1.95 mm). The [18F]FDG PET scans of 112 HC subjects were obtained using either a GE Discovery ST, GE Discovery STE, Siemens Biograph Hi-rez or Siemens ECAT EXACT HR + PET scanner (see (Caminiti et al., 2021, Presotto et al., 2017)). The [18F]FDG-PET acquisition procedures fulfilled the European Association of Nuclear Medicine guidelines (Varrone et al., 2009). Before radiopharmaceutical injection, subjects fasted for at least 6 h to ensure that the measured blood glucose level was <120 mg/dL. Subjects underwent a 3D PET scan (time interval between injection and scan start ranged from 30 to 45 min; scan duration ranged from 10 to 15 min) after the injection of [18F]FDG (185–250 MBq: usually, 5–8 mCi via a venous cannula). Reconstruction of all images was performed using an ordered subset-expectation maximisation algorithm (OSEM, 28 subsets, 2 iterations, 2-mm post-filter). For all clinical groups, CT scans were used for attenuation correction.

### 2.3 Statistical parametric Mapping (SPM) hypometabolism t-maps (Single-subject SPM maps)

Each [18F]FDG-PET image was spatially normalised to a dementia-specific [18F]FDG-PET MNI template (Della Rosa et al., 2014) using SPM12 (https://www.fil.ion.ucl.ac.uk/spm/software/SPM12/) running in MATLAB (MathWorks, Inc., Natick, MA). Images were subsequently smoothed with an isotropic 3D Gaussian kernel (FWHM: 8–8–8 mm). Global mean scaling was applied to each image to account for between-subject uptake variability (Gallivanone et al., 2017). Each pre-proccesed [18F]FDG-PET image was compared to a database of HC (n = 112) (p = 0.05, family-wise error correction at cluster level, 100 voxels of cluster extent) using a previously validated SPM-based method (Caminiti et al., 2021, Perani et al., 2014, Presotto et al., 2017). This SPM-based single-subject procedure was shown not to be influenced by differences in acquisition parameters (i.e. the type of PET camera) between the individual scan and the reference database of HC scans (Caminiti et al., 2021, Presotto et al., 2017).

The resulting hypometabolism t-maps were evaluated by three expert raters (D.P. S.P.C. and G.C.). The aim was to evaluate the presence of metabolic signatures relevant to each disease. For DLB and MSA we referred to the established metabolic features reported in the clinical criteria (Gilman et al., 2008; McKeith et al., 2017). For iRBD and PD, the clinical diagnoses are not based on [18F]FDG-PET outcomes (American Academy of Sleep Medicine, 2014, Postuma et al., 2015). Thus, in these cases, we referred to the previous literature guidelines on the visual rating of SPM t-maps (Carli et al., 2020, Pilotto et al., 2018). iRBD subjects show hypometabolism heterogeneity, presenting in variable portion three SPM t-maps patterns: DLB-like, MSA-like and typical PD-like (Carli et al., 2020). Heterogeneous patterns can be found also in PD patients: typical PD-like, DLB-like, AD-like, frontotemporal dementia- (FTD) like and corticobasal degeneration- (CBD)-like (Pilotto et al., 2018). Thus, raters were forced to decide whether each hypometabolism SPM t-map matched any of the following disease-specific brain metabolic patterns: DLB-like (Caminiti et al., 2019), typical PD-like (Pilotto et al., 2018), MSA-like (Caminiti et al., 2017), AD (Sala et al., 2020), FTD-like (Cerami et al., 2016b) and CBD-like (Caminiti et al., 2017).

The DLB-like pattern was characterised by hypometabolism in the lateral and medial occipital and temporo-parietal cortex, which is variably associated with frontal hypometabolism (Caminiti et al., 2019). The typical PD-like pattern was defined by either no brain hypometabolism or heterogeneity in brain hypometabolism involving motor and premotor regions, somatosensory cortex, anterior cingulate and frontal cortex, and at subcortical level globus pallidus and putamen (Pilotto et al., 2018). The MSA-like pattern consisted of selective either cerebellar or putamen hypometabolism (Caminiti et al., 2017). The atypical patterns corresponded to the following: AD-like patterns, characterised by bilateral temporal-parietal hypometabolism (Sala et al., 2020); CBD-like, with asymmetric frontal-parietal hypometabolism; and FTD-like, with frontal-temporal hypometabolism (Cerami et al., 2016b). Cohen's κ coefficient was used to evaluate the interrater agreement. In case of a disagreement between raters, the most expert rater (D.P.) classification was used for further analyses.

### 2.4 Scaled Subprofile Model/Principal component analysis (SSM/PCA)

In addition to the univariate analyses, we applied an extensively validated multivariate approach in the field of Parkinsonism and related disorders, namely the SSM/PCA (Schindlbeck and Eidelberg, 2018). We aimed to compute the z-scores expression of the main patterns related to the α-synucleinopathies spectrum: PDRP, DLBRP and MSARP. We omitted the RBD-related pattern (RBDRP) because it might represent an early stage of the PDRP (Arnaldi et al., 2019; Meles et al., 2018). Also, the diagnostic and prognostic value of RBDRP in iRBD is still uncertain, since it seems to decrease with the disease progression (Kim et al., 2021).

We used disease-specific spatial covariance patterns (PDRP, MSARP) that were previously identified in cohorts from the University Medical Center Groningen (UMCG) (Meles et al., 2020, Teune et al., 2014, Teune et al., 2013), the Netherlands. We newly identified a DLBRP in a Dutch cohort. We refer to the supplementary material (**S1**) for details.

Subject scores for PDRP, MSARP and DLBRP were calculated in patients and controls. It was shown that acquisition parameters could influence the results in SSM/PCA (Kogan et al., 2019). For this reason, an HC cohort acquired with a similar PET camera and similar acquisition and reconstruction protocols is always needed as a reference. Only 9 of the 112 HC were scanned with a similar camera and protocol (GE Discovery STE) as the patients (iRBD, DLB and MSA were acquired with GE Discovery STE, and PD were acquired with GE Discovery 690) (Table 1).

In the SSM/PCA procedure, images were first log-transformed, and the subject means and group mean (originating from the pattern identification cohort) were removed, resulting in a residual profile for each subject. The subject score was calculated by projecting the subject residual profile on the pattern. Next, subject scores were z-transformed to the subject scores of the 9 HC (Kogan et al., 2019). We applied additional smoothing to PD (GE Discovery 690, N = 28 PD-LDR and N = 16 PD-HDR) and HC (GE Discovery STE, N = 9 patients) images (FWHM: 10–10–10 mm) to minimise the differences between the two GE discovery PET/CT scanners (STE and 690) (Kogan et al., 2019).

### 2.5 Statistical analyses

PDRP, DLBRP and MSARP z-scores were compared across groups using a General Linear Model, controlling for the effect of sex and age. The statistical threshold was set at p < 0.05; a Bonferroni correction was applied for multiple comparisons. The correlations among pattern z-scores were assessed by Pearson correlations in the combined cohort (iRBD, PD-LDR, PD-HDR, DLB and MSA). The diagnostic performance of both methods was determined using ROC curves, using the clinical diagnosis as the reference. For the pattern z-scores, we determined optimal cut-off z-scores with Youden's J statistic (Perkins and Schisterman, 2005).

We performed a group comparison across all six conditions focusing on the differential diagnosis among the overt α-synucleinopathies. The statistical threshold was set at p < 0.05. SPSS 26.0 software and RStudio for Windows were used for statistical analysis.

## 3 Results

Demographics and clinical information are summarised in Table 1. DLB patients were significantly older than PD-LDR and iRBD patients. As expected, DLB patients also had the lowest MMSE scores.

### 3.1 Statistical parametric Mapping (SPM) brain hypometabolism t-maps rating

*Total cohort (iRBD, PD-LDR, PD-HDR, DLB and MSA) –* There was a high interrater agreement in the SPM single-subject classification (Cohen κ >0.90) (McHugh, 2012). According to the rating classification, several hypometabolism patterns emerged at the single-subject level in the total cohort of patients (N = 173). Most of the patterns recognised (92%, N = 159) belonged to the α-synucleinopathies spectrum, i.e., DLB-like, MSA-like, and typical PD-like patterns. Only 8% (N = 14) of patients were classified with patterns related to other neurodegenerative conditions ('atypical patterns'): AD-like, FTD-like, and CBD-like.

*iRBD patients –* The brain hypometabolism patterns in the iRBD group were heterogeneous. The majority was characterised by a DLB-like pattern (N = 31; 61%), followed by typical PD-like patterns (N = 15; 29%) and a minority of cases with MSA-like (N = 5; 10%) patterns.

*PD-LDR patients –* All PD-LDR showed the typical PD-like pattern.

*PD-HDR patients –* showed an equal distribution of DLB-like patterns (44%, N = 7) and atypical patterns (50%, N = 8; 1 CBD-like, 2 FTD-like and 5 AD-like). Only one PD-HDR patient showed a typical PD-like pattern.

*DLB patients* – Most DLB patients showed a DLB-like pattern (N = 61; 91%), and a minority (9%) was characterised by atypical patterns (N = 6, 5 AD-like and 1 CBD-like).

*MSA patients* – All patients showed MSA-like patterns, either MSA-C or MSA-P. Namely, nine MSA patients showed only cerebellar hypometabolism (the patients with the clinical diagnosis of MSA-C), and 2 patients showed selective bilateral putamen hypometabolism (the patients with the clinical diagnosis of MSA-P).

**Fig. 1:**
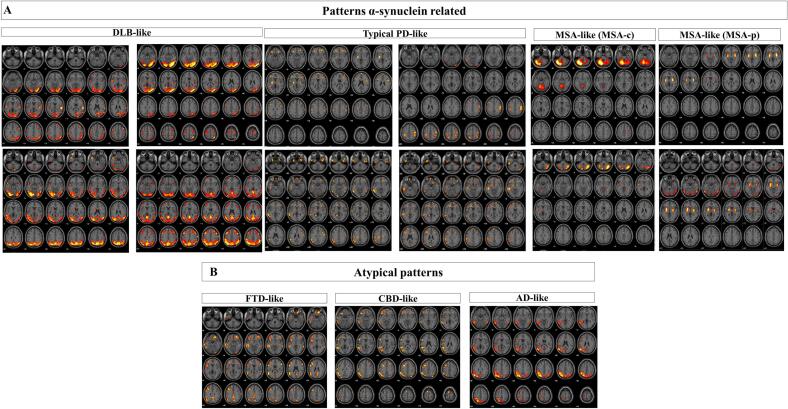
**Examples of α-synuclein-related patterns of neurodegeneration and atypical α-synuclein patterns.** The statistical parametric mapping of [18F]FDG-PET patterns at a single-subject level is depicted for some patients. According to the rating classification, several hypometabolism patterns emerged at the single-subject level in the total cohort of patients (N = 173). Most of the patterns recognised (92%, N = 159) belonged to the α-synucleinopathy spectrum: DLB-like, MSA-like, and typical PD-like patterns. Panel A reports four examples each. Only 8% (14 out of 173) of patients were classified with patterns related to other neurodegenerative conditions ('atypical patterns'): Alzheimer's disease pattern (AD-like), frontotemporal dementia pattern (FTD-like) and corticobasal degeneration pattern (CBD-like). Panel B reports an example each. p < 0.05, FWE corrected at the cluster level.

See Table 2 for the frequencies of patterns across the clinical entities and Fig. 1 for the pattern representations.

**Table 2:** Frequency distribution of neurodegenerative patterns across clinical groups.

	Neurodegeneration Patterns α-synuclein related			Atypical patterns		
	DLB-like	MSA-like	Typical PD-like	AD-like	FTD-like	CBD-like
iRBD (N = 51)	31	5	15^**^	0	0	0
PD-LDR (N = 28)	0	0	28	0	0	0
PD-HDR (N = 16)	7	0	1	5	2	1
DLB (N = 67)	61	0	0	5	0	1
MSA (N = 11)	0	11	0	0	0	0

N = numbers; DLB: Dementia with Lewy Bodies; MSA: Multiple System Atrophy; PD: Parkinson's disease; PD-LDR: PD with low risk of dementia; PD-HDR: PD with high risk of dementia; iRBD: isolated REM sleep behaviour disorder; AD: Alzheimer's disease; FTD: Frontotemporal Dementia; CBD: Corticobasal degeneration.

*15 iRBD showed almost negative SPM single-subject maps. For classification purposes, we included the negative scan under the typical PD-like definition considering the strong topographical similarity. Of course, we cannot exclude the absence of neurodegenerative diseases for iRBD patients see discussion.

### 3.2 Expression of disease-related covariance patterns

**Fig. 2:**
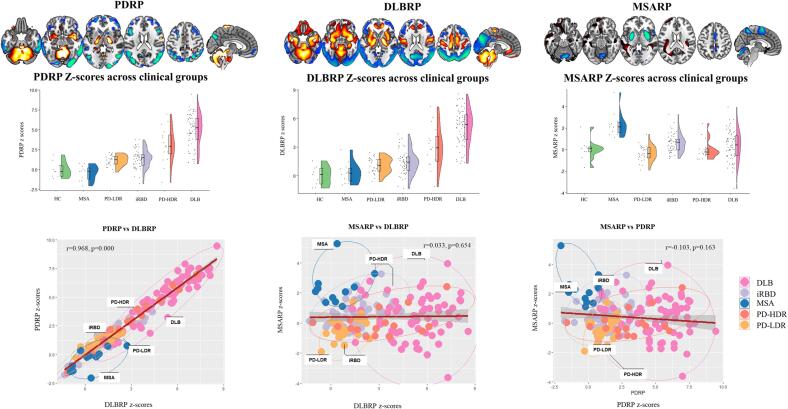
**The topographies of the PDRP, DLBRP and MSARP and the correlations among them.** From top to bottom: the topographies (stable voxels) of the three disease-related metabolic patterns (PDRP, DLBRP, and MSARP), the z-scores expression in each clinical group (violin plots) and Pearson correlations of pattern expression in the combined cohort (scatterplots).

*PDRP –* The PDRP z-scores were significantly different between HC and PD. In PD-LDR, the expression of PDRP (mean ± SD: 1.17±0.67) was higher than in HC (0±1) (p = 0.000), but significantly lower compared to PD-HDR (mean ± SD: 3.07±2.23) (p = 0.000). PDRP z-scores discriminate between HC and PD-LDR (p = 0.003; AUC: 0.837, specificity: 0.777, sensitivity: 0.857 for the optimal cut-off point: 0.53), but the performance was lower than for PD-HDR (p = 0.001; AUC: 0.880, specificity: 0.888, sensitivity: 0.875 for the optimal cut-off point: 1.18) (Fig. 2 and Fig. S1). In PD patients, PDRP z-scores showed a trend towards correlation with UPDRS-III scores (r = 0.29, p = 0.0523) and Rey–Osterrieth complex figure (ROCF)-copy (r = -0.26, p = 0.0933). In DLB patients, PDRP z-scores were correlated with MMSE performances (r = -0.28, p = 0.0447; See Fig. S2).

*DLBRP –* The DLBRP scores were significantly higher in DLB subjects (5.08±1.92) compared with controls (0±1) (P < 0.0001; AUC: 0.947, specificity: 1.000, sensitivity: 0.940 for the optimal cut-off point: 1.62). For details about identification and validation results, see Fig. 2, supplementary materials S1, and Fig. S3. In DLB patients, DLBRP z-scores showed a trend towards correlation with MMSE performances (r = -0.26, p = 0.0607). See Fig. S4.

*MSARP –* In the MSA cohort, MSARP expression (mean ± SD: 2.33±1.16) was higher than in HC (0±1) (p = 0.001; AUC: 0.950, specificity: 0.888, sensitivity: 1.000, for the optimal cut-off point: 0.76) (Fig. 2 and Fig. S5).

#### 3.2.1 Expression of PDRP, DLBRP and MSARP across patient groups

PDRP and DLBRP z-scores were significantly higher in PD-HDR and DLB patients compared with iRBD, PD-LDR and MSA. PDRP and DLBRP z-scores were highly correlated (r = 0.968; P < 0.000). MSARP z-scores were significantly higher in MSA compared with the other diagnoses. MSA patients also displayed the lowest scores for PDRP and DLBRP (Table 3, Fig. 2).

**Table 3:** PDRP, DLBRP and MSARP z-scores across clinical groups.

	iRBD	PD-LDR	PD-HDR	DLB	MSA	p-value	iRBD vs PD-LDR*	iRBD vs PD-HDR*	iRBD vs DLB*	iRBD vs MSA*	PD-LDR vs PD-HDR*	PD-LDR vs DLB*	PD-LDR vs MSA*	PD-HDR vs DLB*	PD-HDR vs MSA*	DLB vs MSA*
PDRP	1.39±1.19	1.17±0.67	3.07±2.23	4.93±2.05	−0.42±0.94	**0.00**	1.00	**0.00**	**0.00**	0.06	**0.00**	**0.00**	0.07	**0.00**	**0.00**	**0.00**
DLBRP	1.41±1.26	1.08±0.81	3.06±2.20	5.08±1.92	0.34±1.11	**0.00**	1.00	**0.00**	**0.00**	1.00	**0.01**	**0.00**	1.00	**0.00**	**0.00**	**0.00**
MSARP	0.61±0.84	−0.35±0.71	0.01±0.91	0.38±1.36	2.33±1.16	**0.00**	**0.01**	**0.01**	1.00	**0.00**	1.00	0.15	**0.00**	1.00	**0.00**	**0.00**

DLB: Dementia with Lewy Bodies; MSA: Multiple System Atrophy; PD-LDR: PD with low risk of dementia; PD-HDR: PD with high risk of dementia; iRBD: isolated REM sleep behaviour disorder.

* p-values Bonferroni corrected.

*iRBD patients* – iRBD patients expressed both PDRP and DLBRP patterns. Their PDRP and DLBRP z-scores were lower than DLB and PD-HDR, but comparable to PD-LDR. These patients showed significantly lower MSARP z-scores (0.61±0.84) than MSA patients (mean ± SD: 2.33±1.16, p = 0.00). However, they showed higher MSARP z-scores than PD-LDR (mean ± SD: −0.35 ± 0.71, p = 0.01) and PD-HDR (mean ± SD: 0.01±0.91, p = 0.01).

*PD-LDR patients –* PD-LDR patients showed a moderate expression of PDRP (mean ± SD: 1.17±0.67), DLBRP (mean ± SD:1.08±0.81) and a low expression of MSARP (mean ± SD: −0.35 ± 0.71). These patients showed a significantly lower expression of PDRP and DLBRP than in DLB (PDRP mean ± SD: 4.93±2.05, p = 0.00; DLBRP mean ± SD: 5.08±1.92, p = 0.00) and in PD-HDR (PDRP mean ± SD: 3.07±2.23, p = 0.00; DLBRP mean ± SD: 3.06±2.2, p = 0.00). They also showed lower MSARP z-scores than in MSA (mean ± SD: 2.33±1.16, p = 0.00) patients.

*PD-HDR patients –* The PD-HDR patients showed high PDRP and DLBRP z-scores, significantly higher than PD-LDR and iRBD, but lower than DLB patients. These patients also showed low MSARP z-scores, significantly lower than MSA and iRBD patients, and comparable to DLB.

*DLB patients –* DLB patients showed the highest PDRP and DLBRP z-scores (PDRP mean ± SD: 4.93±2.05; DLBRP mean ± SD: 5.08±1.9). They also showed significantly lower MSARP z-scores (mean ± SD: 0.38±1.36) compared to MSA patients (mean ± SD: 2.33±1.16, p = 0.00).

*MSA patients –* MSA patients showed the highest MSARP z-scores. These patients showed remarkably low PDRP (mean ± SD: −0.42 ± 0.94) and DLBRP (mean ± SD: 0.34±1.11) z-scores. Although, on average, MSA patients had lower DLBRP z-scores than iRBD (mean ± SD: 1.41±1.26) and PD-LDR (mean ± SD: 1.08±0.81), the statistical threshold did not survive the multiple comparison correction. MSA patients tended to have lower PDRP z-scores than iRBD (mean ± SD: 1.39 ± 1.19, p = 0.06) and PD-LDR (mean ± SD: 1.17 ± 0.67, p = 0.07).

### 3.3 Univariate approach: Diagnostic performances

*iRBD identification –* The SPM single-subject analysis showed low performance in identifying iRBD patients compared to PD-LDR, PD-HDR and DLB (Table S2). When we directly compared iRBD and each clinical group, MSA-like patterns could distinguish between iRBD and MSA (AUC: 0.951; specificity: 1.000; sensitivity: 0.902).

*PD-LDR identification –* The presence of typical PD patterns led to a nearly perfect classification of PD-LDR patients in all tested clinical questions: i) PD-LDR vs. PD-HDR, DLB and MSA (AUC: 0.995; specificity: 0.989; sensitivity: 1.000); ii) PD-LDR vs. PD-HDR and DLB (AUC: 0.994; specificity: 0.988; sensitivity: 1.00) and iv) PD-LDR vs. MSA (AUC: 1.000; specificity: 1.000; sensitivity: 1.000).

*PD-HDR identification –* The SPM single-subject t-maps visual rating provided low classification performance in the two main tested clinical questions: i) PD-HDR vs. PD-LDR, DLB and MSA (DLB-like AUC: 0.569; and atypical patterns: AUC: 0.722); ii) PD-HDR vs. PD-LDR and MSA (DLB-like AUC: 0.719; and atypical patterns: AUC: 0.750).

*DLB identification –* The presence of DLB-like patterns in SPM t-maps showed an optimal performance in identifying DLB patients in most clinical scenarios: i) DLB vs. MSA (AUC: 0.955; specificity: 1.000, sensitivity: 0.910); ii) DLB vs. PD-LDR (AUC: 0.955; specificity: 1.000, sensitivity: 0.910); iii) DLB vs. PD-LDR, PD-HDR and MSA (AUC: 0.892; specificity: 0.872, sensitivity: 0.910). DLB-like patterns showed poor performances in differentiating DLB vs. PD-HDR (AUC: 0.736; specificity: 0.563, sensitivity: 0.910).

*MSA identification –*The presence of the MSA-like pattern using single-subject SPM analysis showed 100% AUC, 1.00 specificity, and 1.00 sensitivity in identifying MSA patients (both MSA-c and MSA-p) compared to the other α-synucleinopathies (PD-LDR, PD-HDR and DLB).

**Fig. 3:**
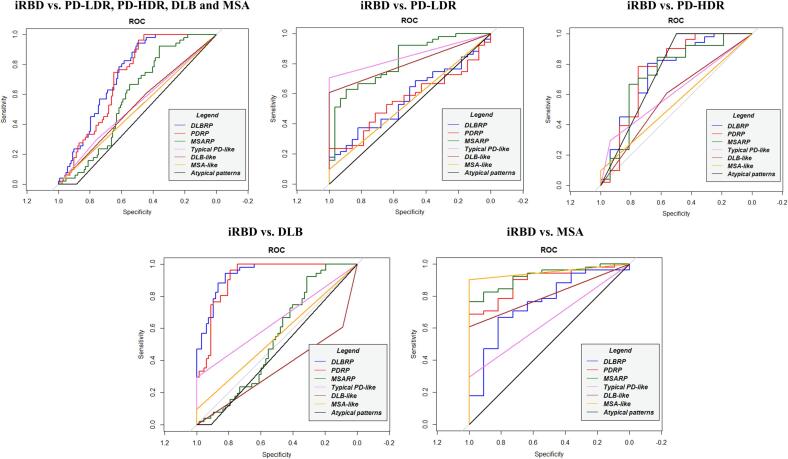
**ROC curves for both methods in iRBD.** Diagnostic accuracy of multivariate and univariate metabolism patterns: DLBRP (blue), PDRP (red), MSARP (green), Typical PD-like pattern (violet), DLB-like pattern (brown), MSA-like pattern (orange) and atypical pattern (black). Abbreviations: iRBD: isolated REM sleep behaviour disorder; PD: Parkison'disease, DLB: Dementia with Lewy Bodies, MSA: Multiple System Atrophy, PD-LDR: PD with low risk of dementia, PD-HDR: PD with high risk of dementia, ROC: Received operating curves; PDRP: PD related pattern; DLBRP: DLB related pattern; MSARP: MSA related pattern. (For interpretation of the references to colour in this figure legend, the reader is referred to the web version of this article.)

**Fig. 4:**
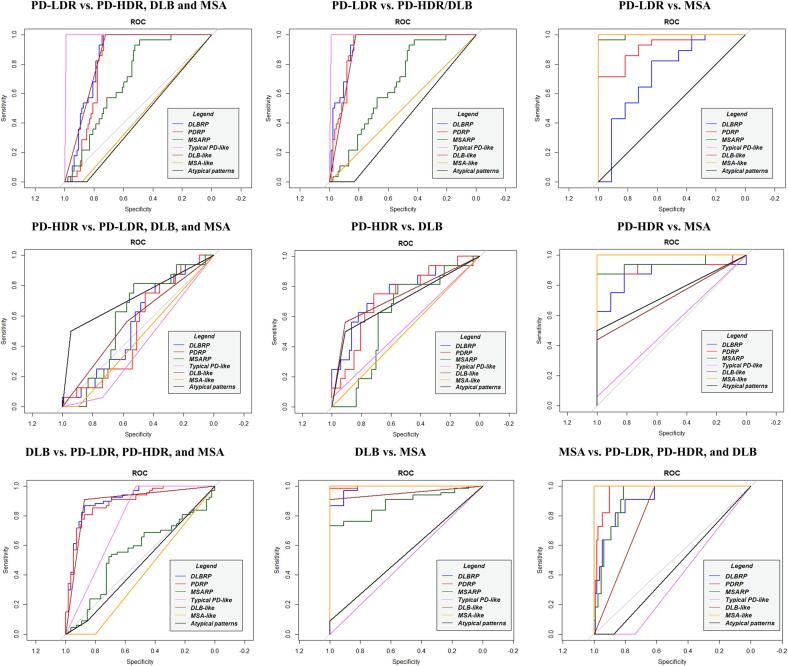
**ROC curves for both methods in overt synucleinopathies.** Diagnostic accuracy of multivariate and univariate metabolism patterns: DLBRP (blue), PDRP (red), MSARP (green), Typical PD-like pattern (violet), DLB-like pattern (brown), MSA-like pattern (orange) and atypical pattern (black). Abbreviations: PD: Parkinson'disease, DLB: Dementia with Lewy Bodies, MSA: Multiple System Atrophy, PD-LDR: PD with low risk of dementia, PD-HDR: PD with high risk of dementia, ROC: Received operating curves; PDRP: PD related pattern; DLBRP: DLB related pattern; MSARP: MSA related pattern. (For interpretation of the references to colour in this figure legend, the reader is referred to the web version of this article.)

For ROC measures and a graphical representation, see Tables S2 – S6 and Fig. 3, Fig. 4.

### 3.4 Multivariate Approach: Diagnostic performances

*iRBD identification* – The SSM/PCA z-scores showed poor performance in identifying iRBD patients compared to the PD-LDR, PD-HDR and MSA (Table S2). However, DLBRP expression could distinguish iRBD from DLB. The threshold of DLBRP z-score > 3.494 correctly differentiated DLB and iRBD (PDRP: AUC: 0.938, specificity: 0.821 and sensitivity: 0.941).

*PD-LDR identification –* PDRP z-scores provided accurate differentiation between PD-LDR and DLB (cut-off: z = 2.21, AUC: 0.940; specificity: 0.851, sensitivity: 1.000). We found sub-optimal performance of the PDRP in terms of specificity comparing PD-LDR vs. PD-HDR (cut-off: z = 2.15; AUC: 0.817; specificity: 0.750, sensitivity: 1.00); and in terms of sensitivity comparing PD-LDR vs. MSA (cut-off: z = 0.782, AUC: 0.919; specificity: 1.000, sensitivity: 0.714).

*PD-HDR identification –* The expression of PDRP provided excellent performance in differentiating PD-HDR and MSA patients (cut-off: 0.977, AUC: 0.926; specificity: 1.000, sensitivity: 0.875). PDRP had sub-optimal performance in the following clinical scenarios: i) PD-HDR vs. DLB (cut-off: z = 4.25, AUC: 0.728; specificity: 0.716, sensitivity: 0.750); ii) PD-HDR vs. PD-LDR (cut-off: z = 2.15, AUC: 0.817; specificity: 1.000, sensitivity: 0.750) and PD-HDR vs DLB, PD-LDR and MSA (cut-off: z = 4.25, AUC: 0.516; specificity: 0.453, sensitivity: 0.750).

*DLB identification –* DLBRP z-scores showed an optimal performance in identifying DLB patients in most clinical scenarios: i) DLB vs. MSA (cut-off: z = 1.46, AUC: 0.985; specificity: 0.909, sensitivity: 0.970); ii) DLB vs. PD-LDR (cut-off: z = 2.42, AUC: 0.962; specificity: 1.000, sensitivity: 0.881); iii) DLB vs. PD-LDR, PD-HDR and MSA (cut-off: z = 3.02, AUC: 0.909; specificity: 0.873, sensitivity: 0.866). DLBRP z-scores showed poor performance in differentiating DLB vs. PD-HDR (cut-off: z = 3.98, AUC: 0.762; specificity: 0.750, sensitivity: 0.716).

*MSA identification –* MSARP z-scores showed high accuracy in identifying MSA patients in all clinical scenarios: i) MSA vs. PD-LDR (cut-off: z = 0.88, AUC: 0.994; specificity: 0.964, sensitivity: 1.000); ii) MSA vs. PD-HDR (cut-off: z = 1.08, AUC: 0.943; specificity: 0.875, sensitivity: 1.000); iii) MSA vs. DLB (cut-off: z = 1.07, AUC: 0.885; specificity: 0.731, sensitivity: 1.000) and iv) MSA vs. PD-LDR, PD-HDR and DLB (cut-off: z = 1.08, AUC: 0.921; specificity: 0.811, sensitivity: 1.000).

For ROC measures and a graphical representation, see Tables S2 – S6 and Fig. 3, Fig. 4.

## 4 Discussion

The early and differential diagnosis in the α-synucleinopathies spectrum is challenging (Brajkovic et al., 2017, Saeed et al., 2017). A shift toward a biological definition of these clinical entities is ongoing, with biomarkers increasingly becoming a relevant part of the diagnostic process. In this context, the role [18F]FDG-PET scans with semi-quantitative methods is well documented (Perani et al., 2020; Schindlbeck and Eidelberg, 2018). Two validated approaches showed promising results in improving the clinical diagnosis of α-synucleinopathies: the SPM single-subject procedure (Della Rosa et al., 2014, Perani et al., 2014) and the SSM/PCA (Eidelberg, 2009, Spetsieris et al., 2013). Both have limitations and strengths.

The SPM single-subject procedure is a univariate approach providing maps at the individual level with the topographical distribution of dysfunctional brain areas. This method improves the qualitative visual rating of [18F]FDG-PET images often used in the clinical diagnostic process, reducing the subjectivity of the rating (Frisoni et al., 2013, Morbelli et al., 2015, Perani et al., 2014). It is important to know that SPM is not the only platform to run this analysis; different advanced parametric tools (also provided by the main PET scanner manufacturers) are available to obtain statistical comparisons between the subjects of interest and a cohort of healthy controls (e.g Neurostat/3D-SSP). This is a strength of the univariate method; it is reproducible with several software and thus easy to apply in routine clinical practice. However, independently from the software used, the single-subject t-maps still require interpretation by an expert with established familiarity with the “metabolic signatures” of neurodegenerative syndromes (Morbelli et al., 2015). Indeed, the main pitfall of this approach is the influence of raters' expertise.

The SSM/PCA approach is a well-established fully quantitative multivariate method with a long history of successful replications of covariance patterns in neurodegenerative diseases, especially PD (Schindlbeck and Eidelberg, 2018). This method identifies specific spatial disease covariance patterns and quantifies their expression within subjects as z-scores. The SSM/PCA approach has enhanced statistical power to detect non-focal changes in imaging signals. However, an important caveat of SSM/PCA concerns harmonisation between scanning platforms and preprocessing algorithms. It has been demonstrated that metabolic brain networks obtained through the SSM/PCA approach are consistent across different reconstruction algorithms (Tomse et al., 2018, Tomše et al., 2017) as long as the z-score computation is done using HC with the same PET camera and reconstruction parameters of patients (Kogan et al., 2019). Thus, the metabolic patterns can be derived in a centre and used in different clinical contexts, favouring multicentric collaborations. However, obtaining an HC cohort is not always feasible and often available only in large, university-affiliated centres. Further harmonisation between scanning platforms and preprocessing algorithms is needed before SSM/PCA can be applied widely in clinical diagnostics (Kogan et al., 2019). On the contrary, these factors do not seem to affect the SPM single-subject procedure (Caminiti et al., 2021, Presotto et al., 2017).

The classification performances of univariate and multivariate methods have been compared in the context of early AD diagnosis (Habeck et al., 2008). Specifically, Habeck and colleagues evaluated the diagnostic performances of AD metabolic markers derived with two methods: voxel-wise group-level comparison between AD and HC (univariate analyses) and AD covariance pattern identified with SSM/PCA (multivariate analyses). Both methods accurately differentiate between AD and controls; however, the SSM/PCA approach benefits from better sensitivity. The authors reported that univariate analyses at the group level fail to capture the individual differences within AD patients, which can cause false negatives. However, this study did not test the diagnostic performance of the SPM single-subject map rating (as a univariate approach), which might overcome this limit.

The present study investigated to what extent the SPM single-subject t-maps or SSM/PCA individual pattern scores (PDRP, DLBRP and MSARP) could distinguish between clinical entities in the α-synucleinopathy spectrum: iRBD, MSA, PD-LDR, PD-HDR and DLB. We found that i) both methods accurately identify PD-LDR patients; however, the rating of SPM t-maps benefit from better sensitivity and specificity, and ii) both methods classified DLB and MSA patients but failed to differentiate PD-HDR and DLB. Of note, we found a gradual increase of PDRP and DLBRP expression in the continuum from iRBD to PD and DLB, where the DLB patients had the highest scores. Only SSM/PCA could differentiate iRBD from DLB, reflecting the differences in disease staging and severity (AUC: 0.938, specificity: 0.821, sensitivity: 0.941). The details of each main finding are discussed in the following paragraphs.

The PD-LDR patients were accurately identified with the rating of SPM t-maps (Table S3). PDRP z-scores also showed good accuracy in identifying PD-LDR patients; however, they had lower sensitivity or specificity than SPM t-maps depending on the optimal cut-off. PD-LDR showed a moderate expression of PDRP, higher than HC and MSA, but lower than PD-HDR and DLB. The optimal cut-off value of 2.15 allowed the discrimination between PD-LDR and PD-HDR, and DLB; this cut-off value was too high to exclude at the same time all MSA patients (false positive), resulting in a lower specificity of the PDPR individual scores (0.734) compared to SPM t-maps (0.989). Applying a lower cut-off value (0.782), PD-LDR and MSA could be accurately differentiated (AUC: 0.919), still with lower sensitivity (0.71) than SPM t-maps (1.000), because of the low expression of PDRP scores of some PD-LDR patients. In any case, PD-LDR and MSA patients could be differentiated by the absence of MSARP expression in PD-LDR. MSARP and PDRP z-scores showed a negative correlation, with two identifiable clusters of patients: i) MSA patients with low PDRP z-scores and high MSARP scores and ii) PD patients with expression of PDRP and low scores of MSARP (Fig. 2). This data highlights the difficulty of establishing an absolute diagnostic threshold for PDRP z-scores. The degree of PDRP expression in PD patients can be variable because it depends on their clinical features and disease stage. In our cohort of PD patients, PDRP z-scores were correlated with motor symptoms (UPDRS-III) and global cognitive functioning (MMSE). This can explain why the PDRP was significantly more expressed in PD-HDR than in PD-LDR. The SPM-based single-subject analysis explained why PD-LDR subjects presented only a moderate expression of PDRP: they showed normal brain metabolism or very limited metabolic involvement of the premotor or motor regions and subcortical structures (Fig. S6) (Pilotto et al., 2018). In addition, they showed considerable variability in the relative hypermetabolism maps (Fig. S6), which did not always resemble the hypermetabolism topography of PDRP (relatively increased activity in the cerebellum, pons, thalamus, putamen/globus pallidus and motor cortex). It might raise the need to define a specific covariance pattern for those PD patients who remain cognitively stable over a long time (PD-LDR). It must be noticed that our study had a specific focus on investigating the role of SPM t-maps and SSM/PCA individual scores in the differential diagnosis within the α-synucleinopathy spectrum. We did not specifically examine the risk of progression to dementia in PD. A more thorough evaluation, including additional measurements, would be needed to assess this risk comprehensively. It is important to acknowledge that several covariance patterns have been identified in PD, such as the PD-related cognitive pattern (PDCP)(Huang et al., 2008, Meles et al., 2015). To fully understand the association between PD and dementia risk, future studies should include quantifying PDCP expression alongside the tested biomarkers. Overall, our findings emphasize the heterogeneity of PD and the challenge of identifying typical brain metabolic patterns related to different phenotypes. Consequently, we think the brain hypometabolism topography at the individual level using SPM t-maps, combined with SSM/PCA individual scores for disease severity/staging measures, is a valuable classification strategy.

Neither method could discriminate accurately between PD-HDR and DLB. This result aligns with the current literature (Caminiti and Carli, 2023). Some consider PDD and DLB as the same clinical disease (Jellinger, 2018), which is supported by neuropathology studies (Ballard et al., 2006, Friedman, 2018, Irwin et al., 2017, Lippa et al., 2007). In the SPM-t map rating, a considerable number of PD-HDR patients had a DLB-like (7/16) pattern, while others (8/16) showed atypical brain hypometabolism patterns (see Fig. 1B and Fig. S7), which can indicate the co-occurrence of other disease-related pathology (Irwin et al., 2013) or misdiagnosis. DLB patients could be identified with SSM/PCA, because they had the highest PDRP and DLBRP z-scores compared to all other clinical diagnoses (total cohort: AUC > 0.90; PD-HDR vs DLB: AUC > 0.70), reflecting the severity of brain metabolic abnormalities in DLB. The “PD-HDR” patients underwent the [18F]FDG-PET scan in their pre-dementia phase (before the dementia diagnosis), which explains why these patients had a lower expression of PDRP and DLBRP compared to established DLB. The PDRP and DLBRP patterns were increasingly expressed along the continuum from iRBD, PD-LDR, PD-HDR and DLB. The degree of pattern expression was indeed previously shown to correlate with disease severity and progression in PD (Schindlbeck and Eidelberg, 2018). Of note, PDRP and DLBRP z-scores showed a very high correlation (r = 0.99, P < 0.0001), and topography overlaps, confirming previous evidence (Lu et al., 2022) (Fig. 2). Shared features between the DLBRP and PDRP include the relative increased metabolism in the subcortical (cerebellum, pons, thalamus, putamen/pallidum) and cortical (sensorimotor cortex) regions, but also hypometabolism of the parietal- and laterofrontal cortex, and especially in the occipital cortex. Both PDRP and DLBRP correlated with cognitive performance in our cohort of PD and DLB patients, suggesting that these patterns might reflect common cognitive degenerative processes. Considering all the above, the strong involvement of the occipital cortex in PDRP topography could mean that its derivation cohort was composed of a considerable number of PD patients in the process of developing a more severe phenotype (PDD). Hypometabolism of the occipital cortex is a well-known signature of DLB (Caminiti et al., 2019) and it is also associated with a faster progression towards dementia in sporadic PD (Pilotto et al., 2018). Indeed, it is related to visuo-spatial, visual-attention, visuo-constructive cognitive impairments and visual hallucinations (Beretta et al., 2019, Firbank et al., 2016, Imamura et al., 1999, Perneczky et al., 2008). Again, these results suggest that PD is a heterogeneous disease with different risks of progression, and the clinical meaning of PDRP obtained from different PD cohorts may vary according to differences in the composition of derivation samples.

Both methods showed high and comparable performance in identifying MSA patients (MSA-C and MSA-P) in the total cohort of αsynucleinopathies (AUC >90). A significant limitation is the small number of MSA patients. In addition, the predominant subtype was MSA-C (9/11 cases), thus requiring confirmation in a larger cohort of MSA-P patients using both methods. The low percentage of MSA-P in our sample seems counterintuitive, considering that MSA-P is more prevalent in Europe (Köllensperger et al., 2010). Our skewed population composition is most likely due to acquisition bias; unfortunately, we cannot confirm this hypothesis. A diagnostic challenge in clinical practice is the early differentiation of MSA-P and PD patients, who often present overlapping clinical features in the early stages. Unfortunately, the data available are currently insufficient to investigate this specific clinical question. However, we found that the SPM procedure fully distinguished MSA-C and MSA-P in single individuals. This suggests that SPM single-subject procedure might represent a useful biomarker to differentiate different phenotypes of MSA.

Lastly, our results also provide some insights into the neurodegenerative mechanisms underlying iRBD. The SPM hypometabolism maps of iRBD already resembled those of full-blown disease (i.e., PD-like, DLB-like and MSA-like), accordingly to previous literature (Carli et al., 2020). This aligns with the evidence that iRBD can variably progress into PD, DLB, PDD and MSA (Galbiati et al., 2019). Specifically, most iRBD (N = 31) showed a DLB-like pattern; SPM single-subject analyses could not differentiate iRBD from DLB and PD-HDR. Only a few (N = 5) patients showed an MSA-like pattern, explaining why MSA and iRBD in a direct comparison could be classified according to the rating of the SPM maps (AUC: 0.951) and MSARP scores (AUC: 0.923). Accordingly, only a limited number of iRBD usually convert into MSA (Galbiati et al., 2019). A portion of iRBD patients also presented normal (typical PD-like) brain metabolism (N = 15), meaning: i) absence of neurodegeneration mechanisms, ii) cases in the process of developing PD without dementia (i.e. PD-LDR) or iii) patients in the earliest stage of the disease who might develop specific patterns later on. Of note, 7 out of these 15 patients already expressed DLBRP and PDRP to some extent (at least one standard deviation higher than HC). This suggests that the SSM/PCA is also very sensitive to early brain changes, capturing the alteration of brain organisation at the beginning of the neurodegenerative process.

At the group level, iRBD expressed higher scores of PDRP and DLBRP than HC (Table 3); however, the z-scores of these patients were also considerably lower than PD-HDR and DLB. We found an increased expression of the two covariance patterns in the continuum from iRBD to PD-HDR and DLB, where the DLB patients expressed the highest scores (Fig. 2). Accordingly, when we tested the classification performance of PDRP and DLBRP in the distinction between iRBD and DLB, they showed good accuracy (AUC: DLBRP: 0.938; PDRP: 0.916, see Table S2). At the same time, although iRBD showed significantly lower z-scores than PD-HDR (Table 3), they could be differentiated sub-optimally (AUC: DLBRP: 0.744; PDRP: 0.759, see Table S2). This might suggest that iRBD, PD-HDR and DLB patients represent different stages of similar neurodegeneration processes. This is in line with the literature on SSM/PCA suggesting that PDRP is a biomarker of disease severity and progression (Arnaldi et al., 2019; Kim et al., 2021, Kogan et al., 2020, Meles et al., 2017). Previously preliminary data reported high PDRP z-scores in iRBD patients close to the point of phenoconversion to PD (Kogan et al., 2020). Our results confirm and expand previous data, highlighting the possible role of the SSM/PCA approach in tracking the disease progression of α-synuclein pathology since the prodromal stages.

This study has some limitations. It is well known that the clinical–pathological diagnosis is the gold standard in α-synucleinopathies (Alafuzoff and Hartikainen, 2018). Thus, the lack of neuropathological data for our cohort of patients represents a limitation. Also, only a small cohort of HC was available as a reference in the SSM/PCA analysis due to the mandatory requirement for this method to include only [18F]FDG-PET scans with a similar acquisition protocol. It must be mentioned that the expression of PDRP, DLBRP and MSARP allowed us to differentiate patients from HC perfectly, validating all the patterns derived in the Netherlands in the here reported cohorts of patients (Figs. S1, S3 and S5). Despite this, the paucity of HC hampered a proper matching for age and gender. Unmatched HC groups might introduce some variance in the z-scoring due to the confounding factors not being controlled (e.g., age and gender). For example, ageing modulates metabolism and having younger HC might result in a higher expression of covariance patterns in patients because of the combined effect of ageing and pathological mechanisms. Most of the patients' groups did not show significant differences in age and gender; thus, they all possibly have a systematic increase of z-scores, most likely not affecting the relationship among patterns expression and, thus, their diagnostic performances. SSM/PCA z-scoring was in high agreement with SPM t-maps, which were corrected for the ageing effect. In addition, the HC and PD cohorts were acquired with different cameras – although the PET cameras were from the same manufacturer. Even if this was accounted for by applying correction strategies, we cannot exclude that this difference influenced the results. In addition, the patients included in this study were retrospectively collected and diagnosed strictly following established diagnostic criteria for each condition, which also can include the [18F]FDG-PET scan. Last, we don't have enough data to replicate the diagnostic performance in independent cohorts of patients, limiting the generalizability of our results.

In conclusion, we suggest that patients' differential diagnosis and prognosis in the α-synucleinopathies spectrum may take advantage of both methods in different situations. The SSM/PCA method provides a rater-independent quantification and is useful for tracking disease progression and even treatment effects (Schindlbeck and Eidelberg, 2018). SSM/PCA provides a scoring that can be interpreted in terms of pattern expression, meaning that it quantifies how much a subject's hypometabolism and hypermetabolism maps correspond to a pre-identified pattern (e.g. PDRP, DLBRP and MSARP). When a subject expresses a low pattern score, we can assume that that subject will not have the topography of the covariance pattern investigated. With the rating of SPM single-subject t-maps, you can verify this assumption by visualising each individual's hyper and hypometabolism topography. This can be particularly helpful when a patient expresses a low pattern value. Our results demonstrate that PD patients have a high variability of hypo- and hypermetabolism patterns at the individual level, explaining why some express moderate scores of PDRP. Thus, the visual rating of SPM t-maps can describe and classify the hypometabolism topography, explaining the intersubjective variability. Inspecting the SPM single-subject maps – revealing the hypometabolic topography at the individual level – is suggested to verify the actual hypometabolism topography distribution.

Our results pave the way for a future combination of univariate and multivariate approaches to improve the diagnosis and prognosis of parkinsonism. Both SPM single-subject and SSM/PCA represent valuable tools. SPM single-subject approach is applied mainly in neuroimaging research and clinical settings with an established role for differential diagnosis and prognosis at the individual level. On the other hand, because the differential diagnosis of neurodegenerative diseases comprises a complex, multi-class problem, SSM/PCA features may be the best with machine learning methods in the future (van Veen et al., 2022).

## Declaration of Competing Interest

The authors declare that they have no known competing financial interests or personal relationships that could have appeared to influence the work reported in this paper.

## Data Availability

The data are available upon reasonable request to the corresponding author according to a formal data-sharing agreement. The data are not publicly available due to privacy/ethical restrictions.
