# Supplementary material for: Comparison of univariate and multivariate analyses for brain [18F]FDG PET data in α-synucleinopathies
Source: Neuroimage Clin. 2023 Jul 13;39:103475. doi: 10.1016/j.nicl.2023.103475 (PMC10394024; doi:10.1016/j.nicl.2023.103475)
Supplement: Supplementary data 1 [file mmc1.docx]

**S1. Identification and Validation of the DLBRP**

**S1.1 Methods**

The DLBRP was identified in a cohort of DLB patients (n=19) and healthy controls (n=19) from the Netherlands (**Table S1**). The 67 Italian DLB patients described in the main text were used for validation.

*DLB cohort The Netherlands -* Nineteen DLB patients with a clinically determined ‘probable’ DLB diagnosis according to consensus criteria [12] were retrospectively included from the out-patient memory clinic and the movement disorders clinic in the University Medical Center Groningen (UMCG), The Netherlands. Standardised assessments included medical history, informant-based history, physical and neurological examination, including Hoehn and Yahr scale, laboratory tests, neuropsychological evaluation, and a structural MRI scan. The neuropsychological screening that was performed was variable: Mini-Mental State Examination (MMSE) was performed on memory clinic patients (n=13), and for patients in the movement disorder clinic the Montreal Cognitive Assessment (MOCA), PD-HDR- short screen [13], or Scales for Outcomes in Parkinson's disease-Cognition (Scopa COG[14]) were applied.

*Identification of DLBRP -* For identification of the DLBRP, we applied an automated algorithm written in-house, based on the SSM PCA method of Spetsieris and Eidelberg [15], implemented in Matlab (version 2017b; MathWorks, Natick, MA). First, a 35% threshold of the whole-brain intensity maximum was applied to each individual [18F]FDG PET image to remove out-of-brain voxels; these were multiplicatively combined to create one common mask that included only non-zero values for all subjects. This mask was applied to all images. Masked images were log-transformed and subject mean and group mean (patients and controls combined) were removed, resulting in a subject residual profile (SRP) per subject. Principal Component Analysis (PCA) was applied in voxel space, and the principal components explaining the top 50% of the total variance were selected for further analysis. For each subject, a score was calculated for each principal component (PC), by projecting the SRP on each PC. Components that gave maximum discrimination between controls and DLB patients (based on these scores) were identified with a stepwise logistic regression procedure, using the lowest Akaike information criterion of the model as a selection criterion [16]. If more than one PC was identified, then these PCs were linearly combined to form one pattern. Each component in this pattern was weighted by the coefficient obtained from the logistic regression model. The final pattern was termed the DLBRP. It is important to note that all voxels in the DLBRP are used for subject score calculation. As indicated above, each subject score was transformed into a z-score with respect to the 19 healthy controls, who were scanned on the same PET camera.

*Validation of DLBRP -* For validation, subject scores for DLBRP were calculated in the Italian cohort (DLB n=67; controls n=9). As indicated above, each subject score was transformed into a z-score with respect to the healthy controls from the same PET camera.

*Visualisation of the DLBRP-* Voxel weights in SSM PCA patterns can fluctuate to some degree depending on the specific sample of patients and controls that is used for derivation [17]. To investigate which regions in each DLBRP were stable, a bootstrap resampling analysis was performed within the derivation set (1000 repetitions). Voxels that survived a two-sided confidence interval (CI) threshold of 90% (5-95%; percentile method) after bootstrapping were overlaid on a T1 MRI template for visualisation. I.e. for positive regions in the pattern, we only displayed those voxels for which the lower bound of the 95% confidence interval is larger than zero. For negative regions in the pattern, we only displayed those voxels for which the upper bound of the 95% confidence interval is smaller than zero.

**S1.2 Results**

The first five PCs explained 51.1% of the total variance in this dataset and were used for further analysis. The first PC (variance explained: 24.9%) could best discriminate between controls and DLB patients in the logistic regression model. The model did not improve with the addition of consecutive, smaller PCs. PC1 was therefore retained and coined the DLBRP. All voxel weights in the DLBRP were overlaid on a T1 MRI template in Montreal Neurological Institute (MNI) space for visualisation (Figure S1.A).

Regions that survived the 90% (5-95%; percentile method) interval after bootstrap resampling are displayed in Figure S2.B. The DLBRP topography is characterised by hypometabolism in the occipital cortex (including the primary visual cortex), parietal cortex and lateral frontal and temporal cortex. Relatively increased metabolism was observed in the brainstem, cerebellum, medial temporal lobe, pallidum, thalamus, orbital and medial frontal cortex, anterior cingulate, and sensorimotor cortex.

DLBRP subject scores are shown in Figure S2.C. DLBRP subject scores were significantly higher in DLB subjects (5.05±1.92) compared with controls (0±1) in the Italian validation cohort (P<0.0001).

**Table S1: Clinical characteristics**

|  | Controls | DLB |
| --- | --- | --- |
| N | 19 | 19 |
| Age in years | 67.2±3.2 | 73.4±6.9^*^ |
| N male (%) | 11 (57) | 16 (84) |
| Disease duration in years† |  | 2±1 |
| H&Y stage |  | 2±1 |
| MMSE |  | 24±1 (n=13) |
| Number of subjects with fluctuations (%) |  | 14 (73) |
| Number of subjects with hallucinations (%) |  | 18 (95) |
| Number of subjects with suspected RBD (%) |  | 10 (63)  (2 unknown) |
| Number of subjects with parkinsonism (%) |  | 17 (89) |
| Duration of clinical follow up in years | NA | 1.9±2.1 |
| *Data are presented with mean and standard deviation, unless otherwise specified.*  *H&Y=Hoehn and Yahr; RBD=REM Sleep behavior disorder; NA = not available or not applicable; MMSE=Mini Mental State Examination*  *^*^Significant difference in age between controls and patients at P<0.01*  *†Disease duration was defined as the time between reported onset of symptoms and the[18F]FDG PET scan* | | |

**Table S2 iRBD identification: ROC curves measures**

| Area Under the Curve: iRBD vs. PD-LDR, PD-HDR, DLB and MSA | | | | | | | | | |  |
| --- | --- | --- | --- | --- | --- | --- | --- | --- | --- | --- |
| Test Result Variable(s) | Area | Asymptotic 95% Confidence Interval | | Optimal Cut-off (Youden Index) | specificity | sensitivity | accuracy | PPV | NPV | Direction |
|  |  | Lower Bound | Upper Bound |  |  |  |  |  |  |  |
| DLBRP | 0.734 | 0.661 | 0.806 | 3.494 | 0.500 | 0.941 | 0.630 | 0.440 | 0.953 | > |
| PDRP | 0.713 | 0.639 | 0.788 | 3.787 | 0.459 | 1.000 | 0.618 | 0.436 | 1.000 | > |
| MSARP | 0.585 | 0.501 | 0.669 | -0.338 | 0.361 | 0.922 | 0.526 | 0.376 | 0.917 | < |
| Typical PD-like pattern | 0.528 | 0.455 | 0.602 | 0.500 | 0.762 | 0.294 | 0.624 | 0.341 | 0.721 | < |
| DLB-like pattern | 0.525 | 0.444 | 0.606 | 0.500 | 0.443 | 0.608 | 0.491 | 0.313 | 0.730 | < |
| MSA-like pattern | 0.504 | 0.455 | 0.552 | 0.500 | 0.910 | 0.098 | 0.671 | 0.313 | 0.707 | < |
| Atypical pattern | 0.443 | 0.414 | 0.471 | - | - | - | - | - | - | < |
| Area Under the Curve: iRBD vs. PD-LDR | | | | | | | | | |  |
| Test Result Variable(s) | Area | Asymptotic 95% Confidence Interval | | Optimal Cut-off (Youden Index) | specificity | sensitivity | accuracy | PPV | NPV | Direction |
|  |  | Lower Bound | Upper Bound | |  |  |  |  |  |  |
| DLBRP | 0.571 | 0.443 | 0.699 | 1.793 | 0.821 | 0.373 | 0.532 | 0.792 | 0.418 | < |
| PDRP | 0.560 | 0.433 | 0.686 | 2.136 | 1.000 | 0.235 | 0.506 | 1.000 | 0.418 | < |
| MSARP | 0.817 | 0.720 | 0.913 | 0.301 | 0.893 | 0.627 | 0.722 | 0.914 | 0.568 | < |
| Typical PD-like pattern | 0.853 | 0.790 | 0.916 | - | 1.000 | 0.706 | 0.810 | 1.000 | 0.651 | > |
| DLB-like pattern | 0.804 | 0.736 | 0.872 | - | 1.000 | 0.608 | 0.747 | 1.000 | 0.583 | < |
| MSA-like pattern | 0.549 | 0.508 | 0.590 | - | 1.000 | 0.098 | 0.418 | 1.000 | 0.378 | < |
| Atypical pattern | 0.500 | 0.500 | 0.500 | - | - | - | - | - | - | < |
| Area Under the Curve: iRBD vs. PD-HDR | | | | | | | | | |  |
| Test Result Variable(s) | Area | Asymptotic 95% Confidence Interval | | Optimal Cut-off (Youden Index) | specificity | sensitivity | accuracy | PPV | NPV | Direction |
|  |  | Lower Bound | Upper Bound | |  |  |  |  |  |  |
| DLBRP | 0.744 | 0.585 | 0.902 | 2.175 | 0.688 | 0.804 | 0.776 | 0.891 | 0.524 | > |
| PDRP | 0.759 | 0.591 | 0.927 | 2.179 | 0.750 | 0.784 | 0.776 | 0.909 | 0.522 | > |
| MSARP | 0.735 | 0.573 | 0.898 | 0.206 | 0.813 | 0.667 | 0.701 | 0.919 | 0.433 | < |
| Typical PD-like pattern | 0.616 | 0.528 | 0.704 | - | 0.938 | 0.294 | 0.448 | 0.938 | 0.294 | < |
| DLB-like pattern | 0.585 | 0.443 | 0.728 | - | 0.563 | 0.608 | 0.597 | 0.816 | 0.310 | < |
| MSA-like pattern | 0.549 | 0.508 | 0.590 | - | 1.000 | 0.098 | 0.313 | 1.000 | 0.258 | < |
| Atypical pattern | 0.750 | 0.623 | 0.877 | - | 0.500 | 1.000 | 0.881 | 0.864 | 1.000 | > |
| Area Under the Curve :iRBD vs. DLB | | | | | | | | | |  |
| Test Result Variable(s) | Area | Asymptotic 95% Confidence Interval | | Optimal Cut-off (Youden Index) | specificity | sensitivity | accuracy | PPV | NPV | Direction |
|  |  | Lower Bound | Upper Bound | |  |  |  |  |  |  |
| DLBRP | 0.938 | 0.899 | 0.977 | 3.494 | 0.821 | 0.941 | 0.873 | 0.800 | 0.948 | > |
| PDRP | 0.916 | 0.866 | 0.966 | 3.189 | 0.791 | 0.961 | 0.864 | 0.778 | 0.964 | > |
| MSARP | 0.536 | 0.432 | 0.641 | -0.351 | 0.313 | 0.922 | 0.576 | 0.505 | 0.840 | < |
| Typical PD-like pattern | 0.647 | 0.584 | 0.710 | - | 1.000 | 0.294 | 0.695 | 1.000 | 0.650 | < |
| DLB-like pattern | 0.349 | 0.273 | 0.425 | - | - | - | - | - | - | < |
| MSA-like pattern | 0.549 | 0.508 | 0.590 | - | 1.000 | 0.098 | 0.610 | 1.000 | 0.593 | < |
| Atypical pattern | 0.455 | 0.421 | 0.490 | - | - | - | - | - | - | < |
| Area Under the Curve: iRBD vs. MSA | | | | | | | | | |  |
| Test Result Variable(s) | Area | Asymptotic 95% Confidence Interval | | Optimal Cut-off (Youden Index) | specificity | sensitivity | accuracy | PPV | NPV | Direction |
|  |  | Lower Bound | Upper Bound | |  |  |  |  |  |  |
| DLBRP | 0.752 | 0.591 | 0.914 | 0.911 | 0.818 | 0.667 | 0.694 | 0.944 | 0.346 | < |
| PDRP | 0.893 | 0.806 | 0.980 | 0.761 | 1.000 | 0.686 | 0.742 | 1.000 | 0.407 | < |
| MSARP | 0.923 | 0.856 | 0.991 | 1.048 | 1.000 | 0.765 | 0.806 | 1.000 | 0.478 | > |
| Typical PD-like pattern | 0.647 | 0.584 | 0.710 | - | 1.000 | 0.294 | 0.419 | 1.000 | 0.234 | < |
| DLB-like pattern | 0.804 | 0.736 | 0.872 | - | 1.000 | 0.608 | 0.677 | 1.000 | 0.355 | < |
| MSA-like pattern | 0.951 | 0.910 | 0.992 | - | 1.000 | 0.902 | 0.919 | 1.000 | 0.688 | > |
| Atypical pattern | 0.500 | 0.500 | 0.500 | - | - | - | - | - | - | < |

**Table S3 PD-LDR identification: ROC curves measures**

| Area Under the Curve: PD-LDR vs. PD-HDR, DLB, and MSA | | | | | | | | | |  |
| --- | --- | --- | --- | --- | --- | --- | --- | --- | --- | --- |
| Test Result Variable(s) | Area | Asymptotic 95% Confidence Interval | | Optimal Cut-off (Youden Index) | specificity | sensitivity | accuracy | PPV | NPV | Direction |
|  |  | Lower Bound | Upper Bound | |  |  |  |  |  |  |
| DLBRP | 0.853 | 0.788 | 0.918 | 2.419 | 0.745 | 1.000 | 0.803 | 0.538 | 1.000 | > |
| PDRP | 0.818 | 0.746 | 0.891 | 2.152 | 0.734 | 1.000 | 0.795 | 0.528 | 1.000 | > |
| MSARP | 0.703 | 0.609 | 0.796 | 0.668 | 0.489 | 0.964 | 0.598 | 0.360 | 0.979 | > |
| Typical PD-like pattern | 0.995 | 0.984 | 1.000 | - | 0.989 | 1.000 | 0.992 | 0.966 | 1.000 | < |
| DLB-like pattern | 0.862 | 0.816 | 0.907 | - | 0.723 | 1.000 | 0.787 | 0.519 | 1.000 | > |
| MSA-like pattern | 0.441 | 0.409 | 0.474 | - | - | - | - | - | - | < |
| Atypical pattern | 0.426 | 0.389 | 0.462 | - | - | - | - | - | - | < |
| Area Under the Curve: PD-LDR vs. PD-HDR | | | | | | | | | |  |
| Test Result Variable(s) | Area | Asymptotic 95% Confidence Interval | | Optimal Cut-off (Youden Index) | specificity | sensitivity | accuracy | PPV | NPV | Direction |
|  |  | Lower Bound | Upper Bound | |  |  |  |  |  |  |
| DLBRP | 0.804 | 0.640 | 0.967 | 2.585 | 0.625 | 1.000 | 0.864 | 0.824 | 1.000 | > |
| PDRP | 0.817 | 0.644 | 0.990 | 2.152 | 0.750 | 1.000 | 0.909 | 0.875 | 1.000 | > |
| MSARP | 0.578 | 0.403 | 0.753 | -0.443 | 0.750 | 0.464 | 0.568 | 0.765 | 0.444 | > |
| Typical PD-like pattern | 0.969 | 0.908 | 1.000 | - | 0.938 | 1.000 | 0.977 | 0.966 | 1.000 | < |
| DLB-like pattern | 0.281 | 0.156 | 0.407 | - | - | - | - | - | - | < |
| MSA-like pattern | 0.500 | 0.500 | 0.500 | - | - | - | - | - | - | < |
| Atypical pattern | 0.750 | 0.623 | 0.877 | - | 0.500 | 1.000 | 0.818 | 0.778 | 1.000 | > |
| Area Under the Curve: PD-LDR vs. DLB | | | | | | | | | |  |
| Test Result Variable(s) | Area | Asymptotic 95% Confidence Interval | | Optimal Cut-off (Youden Index) | specificity | sensitivity | accuracy | PPV | NPV | Direction |
|  |  | Lower Bound | Upper Bound | |  |  |  |  |  |  |
| DLBRP | 0.962 | 0.930 | 0.994 | 2.419 | 0.881 | 1.000 | 0.916 | 0.778 | 1.000 | > |
| PDRP | 0.940 | 0.895 | 0.985 | 2.213 | 0.851 | 1.000 | 0.895 | 0.737 | 1.000 | > |
| MSARP | 0.684 | 0.579 | 0.789 | 0.385 | 0.522 | 0.929 | 0.642 | 0.448 | 0.946 | > |
| Typical PD-like pattern | 1.000 | 1.000 | 1.000 | - | 1.000 | 1.000 | 1.000 | 1.000 | 1.000 | < |
| DLB-like pattern | 0.955 | 0.921 | 0.990 | - | 0.910 | 1.000 | 0.937 | 0.824 | 1.000 | > |
| MSA-like pattern | 0.500 | 0.500 | 0.500 | - | - | - | - | - | - | < |
| Atypical pattern | 0.455 | 0.421 | 0.490 | - | - | - | - | - | - | < |
| Area Under the Curve: PD-LDR vs. PD-HDR and DLB | | | | | | | | | |  |
| Test Result Variable(s) | Area | Asymptotic 95% Confidence Interval | | Optimal Cut-off (Youden Index) | specificity | sensitivity | accuracy | PPV | NPV | Direction |
|  |  | Lower Bound | Upper Bound | |  |  |  |  |  |  |
| DLBRP | 0.932 | 0.887 | 0.976 | 2.419 | 0.831 | 1.000 | 0.874 | 0.667 | 1.000 | > |
| PDRP | 0.916 | 0.865 | 0.967 | 2.152 | 0.831 | 1.000 | 0.874 | 0.667 | 1.000 | > |
| MSARP | 0.664 | 0.561 | 0.767 | 0.385 | 0.458 | 0.929 | 0.577 | 0.366 | 0.950 | > |
| Typical PD-like pattern | 0.994 | 0.982 | 1.000 | - | 0.988 | 1.000 | 0.991 | 0.966 | 1.000 | < |
| DLB-like pattern | 0.910 | 0.868 | 0.951 | - | 0.819 | 1.000 | 0.865 | 0.651 | 1.000 | > |
| MSA-like pattern | 0.500 | 0.500 | 0.500 | - | - | - | - | - | - | < |
| Atypical pattern | 0.416 | 0.375 | 0.456 | - | - | - | - | - | - | < |
| Area Under the Curve: PD-LDR vs. MSA | | | | | | | | | |  |
| Test Result Variable(s) | Area | Asymptotic 95% Confidence Interval | | Optimal Cut-off (Youden Index) | specificity | sensitivity | accuracy | PPV | NPV | Direction |
|  |  | Lower Bound | Upper Bound | |  |  |  |  |  |  |
| DLBRP | 0.737 | 0.537 | 0.937 | 0.384 | 0.636 | 0.821 | 0.769 | 0.852 | 0.583 | < |
| PDRP | 0.919 | 0.833 | 1.000 | 0.782 | 1.000 | 0.714 | 0.795 | 1.000 | 0.579 | < |
| MSARP | 0.994 | 0.978 | 1.000 | 0.875 | 1.000 | 0.964 | 0.974 | 1.000 | 0.917 | > |
| Typical PD-like pattern | 1.000 | 1.000 | 1.000 | - | 1.000 | 1.000 | 1.000 | 1.000 | 1.000 | < |
| DLB-like pattern | 0.500 | 0.500 | 0.500 | - | - | - | - | - | - | < |
| MSA-like pattern | 1.000 | 1.000 | 1.000 | - | 1.000 | 1.000 | 1.000 | 1.000 | 1.000 | > |
| Atypical pattern | 0.500 | 0.500 | 0.500 | - | - | - | - | - | - | < |

**Table S4 PD-HDR identification: ROC curves measures**

| Area Under the Curve: PD-HDR vs. PD-LDR, DLB, and MSA | | | | | | | | | |  |
| --- | --- | --- | --- | --- | --- | --- | --- | --- | --- | --- |
| Test Result Variable(s) | Area | Asymptotic 95% Confidence Interval | | Optimal Cut-off (Youden Index) | specificity | sensitivity | accuracy | PPV | NPV | Direction |
|  |  | Lower Bound | Upper Bound | |  |  |  |  |  |  |
| DLBRP | 0.546 | 0.410 | 0.682 | 3.978 | 0.453 | 0.750 | 0.492 | 0.171 | 0.923 | > |
| PDRP | 0.516 | 0.382 | 0.650 | 4.255 | 0.453 | 0.750 | 0.492 | 0.171 | 0.923 | > |
| MSARP | 0.591 | 0.462 | 0.719 | 0.191 | 0.528 | 0.813 | 0.566 | 0.206 | 0.949 | > |
| Typical PD-like pattern | 0.399 | 0.325 | 0.474 | - | - | - | - | - | - | < |
| DLB-like pattern | 0.569 | 0.435 | 0.703 | - | 0.575 | 0.563 | 0.574 | 0.167 | 0.897 | > |
| MSA-like pattern | 0.448 | 0.419 | 0.477 | - | - | - | - | - | - | < |
| Atypical pattern | 0.722 | 0.593 | 0.850 | - | 0.943 | 0.500 | 0.885 | 0.571 | 0.926 | < |
| Area Under the Curve: PD-HDR vs. PD-LDR and MSA | | | | | | | | | |  |
| Test Result Variable(s) | Area | Asymptotic 95% Confidence Interval | | Optimal Cut-off (Youden Index) | specificity | sensitivity | accuracy | PPV | NPV | Direction |
|  |  | Lower Bound | Upper Bound | |  |  |  |  |  |  |
| DLBRP | 0.825 | 0.675 | 0.976 | 2.738 | 1.000 | 0.625 | 0.891 | 1.000 | 0.867 | < |
| PDRP | 0.848 | 0.697 | 0.999 | 2.152 | 1.000 | 0.750 | 0.927 | 1.000 | 0.907 | < |
| MSARP | 0.569 | 0.413 | 0.725 | 0.191 | 0.487 | 0.813 | 0.582 | 0.394 | 0.864 | > |
| Typical PD-like pattern | 0.828 | 0.734 | 0.922 | - | 0.718 | 0.938 | 0.782 | 0.577 | 0.966 | > |
| DLB-like pattern | 0.719 | 0.593 | 0.844 | - | 1.000 | 0.438 | 0.836 | 1.000 | 0.813 | < |
| MSA-like pattern | 0.359 | 0.287 | 0.431 | - | - | - | - | - | - | < |
| Atypical pattern | 0.750 | 0.624 | 0.877 | - | 1.000 | 0.500 | 0.855 | 1.000 | 0.830 | < |
| Area Under the Curve: PD-HDR vs. DLB | | | | | | | | | |  |
| Test Result Variable(s) | Area | Asymptotic 95% Confidence Interval | | Optimal Cut-off (Youden Index) | specificity | sensitivity | accuracy | PPV | NPV | Direction |
|  |  | Lower Bound | Upper Bound | |  |  |  |  |  |  |
| DLBRP | 0.7621 | 0.623 | 0.902 | 3.978 | 0.716 | 0.750 | 0.723 | 0.387 | 0.923 | > |
| PDRP | 0.728 | 0.592 | 0.864 | 4.255 | 0.716 | 0.750 | 0.723 | 0.387 | 0.923 | > |
| MSARP | 0.604 | 0.463 | 0.745 | 0.204 | 0.552 | 0.813 | 0.602 | 0.302 | 0.925 | > |
| Typical PD-like pattern | 0.531 | 0.470 | 0.593 | - | 1.000 | 0.063 | 0.819 | 1.000 | 0.817073 | < |
| DLB-like pattern | 0.737 | 0.606 | 0.867 | - | 0.910 | 0.563 | 0.843 | 0.600 | 0.897 | > |
| MSA-like pattern | 0.500 | 0.500 | 0.500 | - | - | - | - | - | - | < |
| Atypical pattern | 0.705 | 0.574 | 0.836 | - | 0.910 | 0.500 | 0.831 | 0.571 | 0.884 | < |
| Area Under the Curve: PD-HDR vs. MSA | | | | | | | | | |  |
| Test Result Variable(s) | Area | Asymptotic 95% Confidence Interval | | Optimal Cut-off (Youden Index) | specificity | sensitivity | accuracy | PPV | NPV | Direction |
|  |  | Lower Bound | Upper Bound | |  |  |  |  |  |  |
| DLBRP | 0.881 | 0.741 | 1.000 | 1.043 | 0.818 | 0.875 | 0.852 | 0.875 | 0.818 | < |
| PDRP | 0.926 | 0.810 | 1.000 | 0.977 | 1.000 | 0.875 | 0.926 | 1.000 | 0.846 | < |
| MSARP | 0.943 | 0.849 | 1.000 | 1.088 | 1.000 | 0.875 | 0.926 | 1.000 | 0.846 | > |
| Typical PD-like pattern | 0.531 | 0.470 | 0.593 | - | 1.000 | 0.063 | 0.444 | 1.000 | 0.423077 | < |
| DLB-like pattern | 0.719 | 0.593 | 0.844 | - | 1.000 | 0.438 | 0.667 | 1.000 | 0.550 | < |
| MSA-like pattern | 1.000 | 1.000 | 1.000 | - | 1.000 | 1.000 | 1.000 | 1.000 | 1.000 | > |
| Atypical pattern | 0.750 | 0.624 | 0.877 | - | 1.000 | 0.500 | 0.704 | 1.000 | 0.578947 | < |
| Area Under the Curve: PD-HDR vs. PD-LDR | | | | | | | | | |  |
| Test Result Variable(s) | Area | Asymptotic 95% Confidence Interval | | Optimal Cut-off (Youden Index) | specificity | sensitivity | accuracy | PPV | NPV | Direction |
|  |  | Lower Bound | Upper Bound | |  |  |  |  |  |  |
| DLBRP | 0.804 | 0.640 | 0.967 | 2.585 | 1.000 | 0.625 | 0.864 | 1.000 | 0.824 | < |
| PDRP | 0.817 | 0.644 | 0.990 | 2.152 | 1.000 | 0.750 | 0.909 | 1.000 | 0.875 | < |
| MSARP | 0.578 | 0.403 | 0.753 | -0.443 | 0.464 | 0.750 | 0.568 | 0.444 | 0.765 | < |
| Typical PD-like pattern | 0.969 | 0.908 | 1.000 | - | 1.000 | 0.938 | 0.977 | 1.000 | 0.966 | > |
| DLB-like pattern | 0.719 | 0.593 | 0.844 | - | 1.000 | 0.438 | 0.795 | 1.000 | 0.757 | < |
| MSA-like pattern | 0.500 | 0.500 | 0.500 | - | - | - | - | - | - | < |
| Atypical pattern | 0.750 | 0.624 | 0.877 | - | 1.000 | 0.500 | 0.818 | 1.000 | 0.778 | < |

**Table S5 DLB identification: ROC curves measures**

| Area Under the Curve: DLB vs. PD-LDR, PD-HDR, and MSA | | | | | | | | | |  |
| --- | --- | --- | --- | --- | --- | --- | --- | --- | --- | --- |
| Test Result Variable(s) | Area | Asymptotic 95% Confidence Interval | | Optimal Cut-off (Youden Index) | specificity | sensitivity | accuracy | PPV | NPV | Direction |
|  |  | Lower Bound | Upper Bound |  |  |  |  |  |  |  |
| DLBRP | 0.909 | 0.854 | 0.963 | 3.028 | 0.873 | 0.866 | 0.869 | 0.892 | 0.842 | < |
| PDRP | 0.890 | 0.831 | 0.948 | 3.014 | 0.873 | 0.806 | 0.836 | 0.885 | 0.787 | < |
| MSARP | 0.547 | 0.442 | 0.652 | 0.385 | 0.709 | 0.522 | 0.607 | 0.686 | 0.549 | < |
| Typical PD-like pattern | 0.764 | 0.697 | 0.830 | - | 0.527 | 1.000 | 0.787 | 0.720 | 1.000 | > |
| DLB-like pattern | 0.892 | 0.835 | 0.948 | - | 0.872 | 0.910 | 0.893 | 0.897 | 0.889 | < |
| MSA-like pattern | 0.400 | 0.347 | 0.453 | - | - | - | - | - | - | < |
| Atypical pattern | 0.472 | 0.414 | 0.530 | - | - | - | - | - | - | < |
| Area Under the Curve: DLB vs. PD-LDR and MSA | | | | | | | | | |  |
| Test Result Variable(s) | Area | Asymptotic 95% Confidence Interval | | Optimal Cut-off (Youden Index) | specificity | sensitivity | accuracy | PPV | NPV | Direction |
|  |  | Lower Bound | Upper Bound | |  |  |  |  |  |  |
| DLBRP | 0.969 | 0.942 | 0.995 | 2.884 | 1.000 | 0.866 | 0.915 | 1.000 | 0.813 | < |
| PDRP | 0.956 | 0.922 | 0.990 | 2.213 | 1.000 | 0.851 | 0.906 | 1.000 | 0.796 | < |
| MSARP | 0.524 | 0.407 | 0.641 | 0.385 | 0.667 | 0.522 | 0.575 | 0.729 | 0.448 | < |
| Typical PD-like pattern | 0.859 | 0.787 | 0.931 | - | 0.718 | 1.000 | 0.896 | 0.859 | 1.000 | > |
| DLB-like pattern | 0.955 | 0.921 | 0.990 | - | 1.000 | 0.910 | 0.943 | 1.000 | 0.867 | < |
| MSA-like pattern | 0.359 | 0.287 | 0.431 | - | - | - | - | - | - | < |
| Atypical pattern | 0.545 | 0.510 | 0.579 | - | 1.000 | 0.090 | 0.425 | 1.000 | 0.390 | < |
| Area Under the Curve: DLB vs. PD-HDR | | | | | | | | | |  |
| Test Result Variable(s) | Area | Asymptotic 95% Confidence Interval | | Optimal Cut-off (Youden Index) | specificity | sensitivity | accuracy | PPV | NPV | Direction |
|  |  | Lower Bound | Upper Bound | |  |  |  |  |  |  |
| DLBRP | 0.762 | 0.623 | 0.902 | 3.978 | 0.750 | 0.716 | 0.723 | 0.923 | 0.387 | < |
| PDRP | 0.728 | 0.591 | 0.864 | 4.255 | 0.750 | 0.716 | 0.723 | 0.923 | 0.387 | < |
| MSARP | 0.604 | 0.463 | 0.745 | 0.204 | 0.813 | 0.552 | 0.602 | 0.925 | 0.302 | < |
| Typical PD-like pattern | 0.469 | 0.408 | 0.530 | - | - | - | - | - | - | < |
| DLB-like pattern | 0.736 | 0.606 | 0.867 | - | 0.563 | 0.910 | 0.843 | 0.897 | 0.600 | < |
| MSA-like pattern | 0.500 | 0.500 | 0.500 | - | - | - | - | - | - | < |
| Atypical pattern | 0.705 | 0.574 | 0.836 | - | 0.500 | 0.910 | 0.831 | 0.884 | 0.571 | > |
| Area Under the Curve: DLB vs. MSA | | | | | | | | | |  |
| Test Result Variable(s) | Area | Asymptotic 95% Confidence Interval | | Optimal Cut-off (Youden Index) | specificity | sensitivity | accuracy | PPV | NPV | Direction |
|  |  | Lower Bound | Upper Bound | |  |  |  |  |  |  |
| DLBRP | 0.985 | 0.959 | 1.000 | 1.467 | 0.909 | 0.970 | 0.962 | 0.985 | 0.833 | < |
| PDRP | 0.997 | 0.991 | 1.000 | 0.807 | 1.000 | 0.985 | 0.987 | 1.000 | 0.917 | < |
| MSARP | 0.885 | 0.806 | 0.964 | 1.078 | 1.000 | 0.731 | 0.769 | 1.000 | 0.379 | > |
| Typical PD-like pattern | 0.500 | 0.500 | 0.500 | - | - | - | - | - | - | < |
| DLB-like pattern | 0.955 | 0.921 | 0.990 | - | 1.000 | 0.910 | 0.923 | 1.000 | 0.647 | < |
| MSA-like pattern | 1.000 | 1.000 | 1.000 | - | 1.000 | 1.000 | 1.000 | 1.000 | 1.000 | > |
| Atypical pattern | 0.545 | 0.510 | 0.579 | - | 1.000 | 0.090 | 0.218 | 1.000 | 0.153 | < |
| Area Under the Curve: DLB vs. PD-LDR | | | | | | | | | |  |
| Test Result Variable(s) | Area | Asymptotic 95% Confidence Interval | | Optimal Cut-off (Youden Index) | specificity | sensitivity | accuracy | PPV | NPV | Direction |
|  |  | Lower Bound | Upper Bound | |  |  |  |  |  |  |
| DLBRP | 0.962 | 0.930 | 0.994 | 2.419 | 1.000 | 0.881 | 0.916 | 1.000 | 0.778 | < |
| PDRP | 0.940 | 0.895 | 0.985 | 2.213 | 1.000 | 0.851 | 0.895 | 1.000 | 0.737 | < |
| MSARP | 0.684 | 0.579 | 0.789 | 0.385 | 0.929 | 0.522 | 0.642 | 0.946 | 0.448 | < |
| Typical PD-like pattern | 1.000 | 1.000 | 1.000 | - | 1.000 | 1.000 | 1.000 | 1.000 | 1.000 | > |
| DLB-like pattern | 0.955 | 0.921 | 0.990 | - | 1.000 | 0.910 | 0.937 | 1.000 | 0.824 | < |
| MSA-like pattern | 0.500 | 0.500 | 0.500 | - | - | - | - | - | - | < |
| Atypical pattern | 0.545 | 0.510 | 0.579 | - | 1.000 | 0.090 | 0.358 | 1.000 | 0.315 | < |

**Table S6 MSA identification: ROC curves measures**

| Area Under the Curve: MSA vs. PD-LDR, PD-HDR, and DLB | | | | | | | | | |  |
| --- | --- | --- | --- | --- | --- | --- | --- | --- | --- | --- |
| Test Result Variable(s) | Area | Asymptotic 95% Confidence Interval | | Optimal Cut-off (Youden Index) | specificity | sensitivity | accuracy | PPV | NPV | Direction |
|  |  | Lower Bound | Upper Bound |  |  |  |  |  |  |  |
| DLBRP | 0.907 | 0.832 | 0.983 | 1.467 | 0.802 | 0.909 | 0.811 | 0.313 | 0.989 | > |
| PDRP | 0.967 | 0.936 | 0.998 | 0.782 | 0.901 | 1.000 | 0.910 | 0.500 | 1.000 | > |
| MSARP | 0.921 | 0.867 | 0.974 | 1.088 | 0.811 | 1.000 | 0.828 | 0.344 | 1.000 | < |
| Typical PD-like pattern | 0.369 | 0.328 | 0.410 | - | - | - | - | - | - | < |
| DLB-like pattern | 0.806 | 0.761 | 0.852 | - | 0.613 | 1.000 | 0.648 | 0.204 | 1.000 | > |
| MSA-like pattern | 1.000 | 1.000 | 1.000 | - | 1.000 | 1.000 | 1.000 | 1.000 | 1.000 | < |
| Atypical pattern | 0.437 | 0.406 | 0.468 | - | - | - | - | - | - | < |
| Area Under the Curve: MSA vs. PD-HDR | | | | | | | | | |  |
| Test Result Variable(s) | Area | Asymptotic 95% Confidence Interval | | Optimal Cut-off (Youden Index) | specificity | sensitivity | accuracy | PPV | NPV | Direction |
|  |  | Lower Bound | Upper Bound | |  |  |  |  |  |  |
| DLBRP | 0.881 | 0.741 | 1.000 | 1.043 | 0.875 | 0.818 | 0.852 | 0.818 | 0.875 | > |
| PDRP | 0.926 | 0.810 | 1.000 | 0.977 | 0.875 | 1.000 | 0.926 | 0.846 | 1.000 | > |
| MSARP | 0.943 | 0.849 | 1.000 | 1.088 | 0.875 | 1.000 | 0.926 | 0.846 | 1.000 | < |
| Typical PD-like pattern | 0.469 | 0.408 | 0.530 | - | - | - | - | - | - | < |
| DLB-like pattern | 0.281 | 0.156 | 0.407 | - | - | - | - | - | - | < |
| MSA-like pattern | 1.000 | 1.000 | 1.000 | - | 1.000 | 1.000 | 1.000 | 1.000 | 1.000 | < |
| Atypical pattern | 0.750 | 0.623 | 0.877 | - | 0.500 | 1.000 | 0.704 | 0.579 | 1.000 | > |
| Area Under the Curve: MSA vs. DLB | | | | | | | | | |  |
| Test Result Variable(s) | Area | Asymptotic 95% Confidence Interval | | Optimal Cut-off (Youden Index) | specificity | sensitivity | accuracy | PPV | NPV | Direction |
|  |  | Lower Bound | Upper Bound | |  |  |  |  |  |  |
| DLBRP | 0.985 | 0.959 | 1.000 | 1.467 | 0.970 | 0.909 | 0.962 | 0.833 | 0.985 | > |
| PDRP | 0.997 | 0.991 | 1.000 | 0.807 | 0.985 | 1.000 | 0.987 | 0.917 | 1.000 | > |
| MSARP | 0.885 | 0.806 | 0.964 | 1.078 | 0.731 | 1.000 | 0.769 | 0.379 | 1.000 | < |
| Typical PD-like pattern | 0.500 | 0.500 | 0.500 | - | - | - | - | - | - | < |
| DLB-like pattern | 0.955 | 0.921 | 0.990 | - | 0.910 | 1.000 | 0.923 | 0.647 | 1.000 | > |
| MSA-like pattern | 1.000 | 1.000 | 1.000 | - | 1.000 | 1.000 | 1.000 | 1.000 | 1.000 | < |
| Atypical pattern | 0.455 | 0.421 | 0.490 | - | - | - | - | - | - | < |
| Area Under the Curve: MSA vs. PD-LDR | | | | | | | | | |  |
| Test Result Variable(s) | Area | Asymptotic 95% Confidence Interval | | Optimal Cut-off (Youden Index) | specificity | sensitivity | accuracy | PPV | NPV | Direction |
|  |  | Lower Bound | Upper Bound | |  |  |  |  |  |  |
| DLBRP | 0.737 | 0.537 | 0.937 | 0.384 | 0.821 | 0.636 | 0.769 | 0.583 | 0.852 | > |
| PDRP | 0.919 | 0.833 | 1.000 | 0.782 | 0.714 | 1.000 | 0.795 | 0.579 | 1.000 | > |
| MSARP | 0.994 | 0.978 | 1.000 | 0.875 | 0.964 | 1.000 | 0.974 | 0.917 | 1.000 | < |
| Typical PD-like pattern | 1.000 | 1.000 | 1.000 | - | 1.000 | 1.000 | 1.000 | 1.000 | 1.000 | > |
| DLB-like pattern | 0.500 | 0.500 | 0.500 | - | - | - | - | - | - | < |
| MSA-like pattern | 1.000 | 1.000 | 1.000 | - | 1.000 | 1.000 | 1.000 | 1.000 | 1.000 | < |
| Atypical pattern | 0.500 | 0.500 | 0.500 | - | - | - | - | - | - | < |


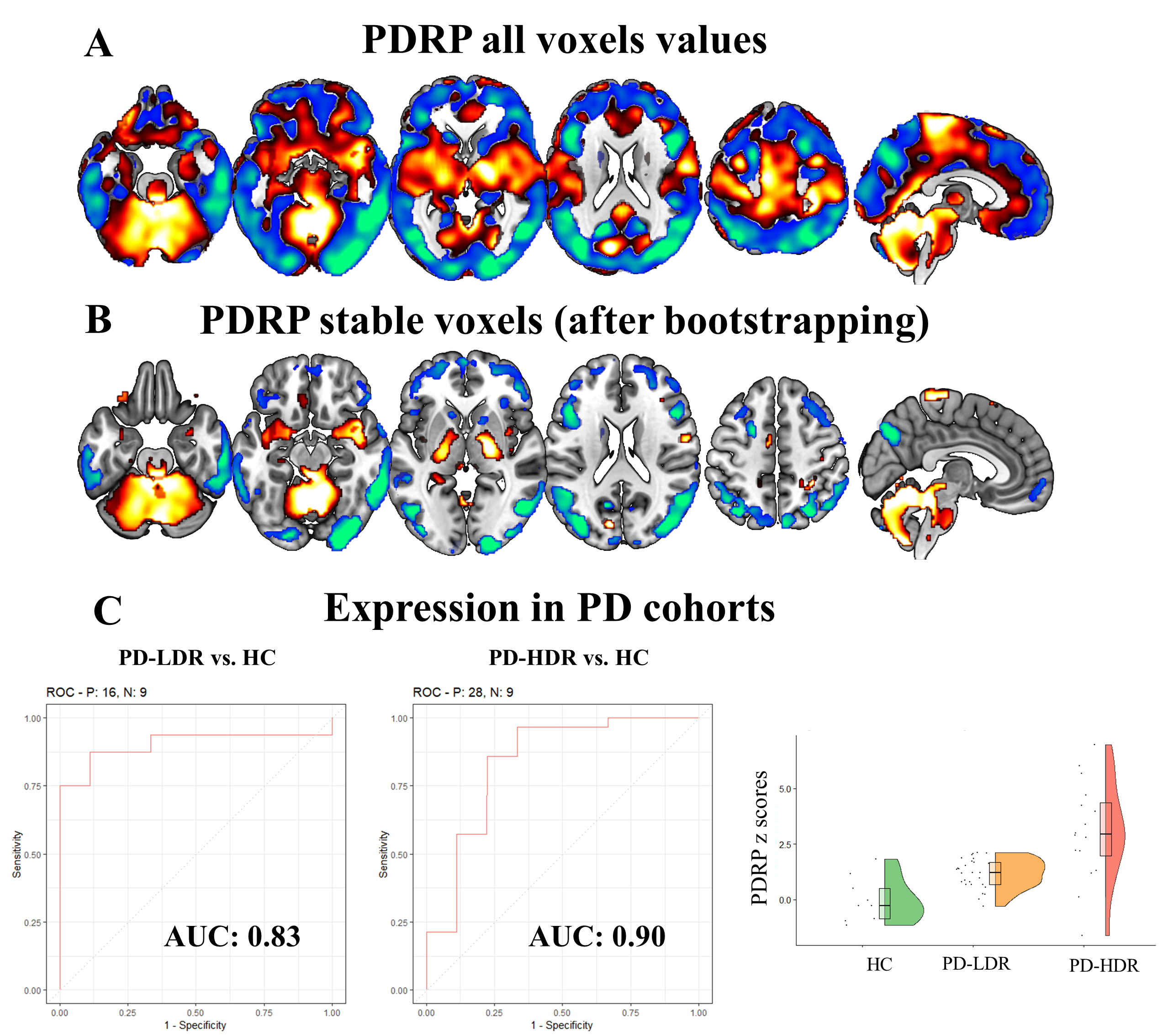


**Figure S1** **PDRP topography and validation in the Italian cohort.** All voxel (A) and stable values (B) are overlaid on a T1 MR template. The discrimination performance and z-scores expressions in the validation (IT) cohort is represented throughout ROCs curves and raincloud graphs, respectively (C). Abbreviations: PDRP: Parkinson’s disease-related pattern; HC: healthy controls; PD-LDR: PD with normal cognition over time, PD-HDR: Parkinson’s disease with dementia. AUC: Area Under the Curve; vs: versus.


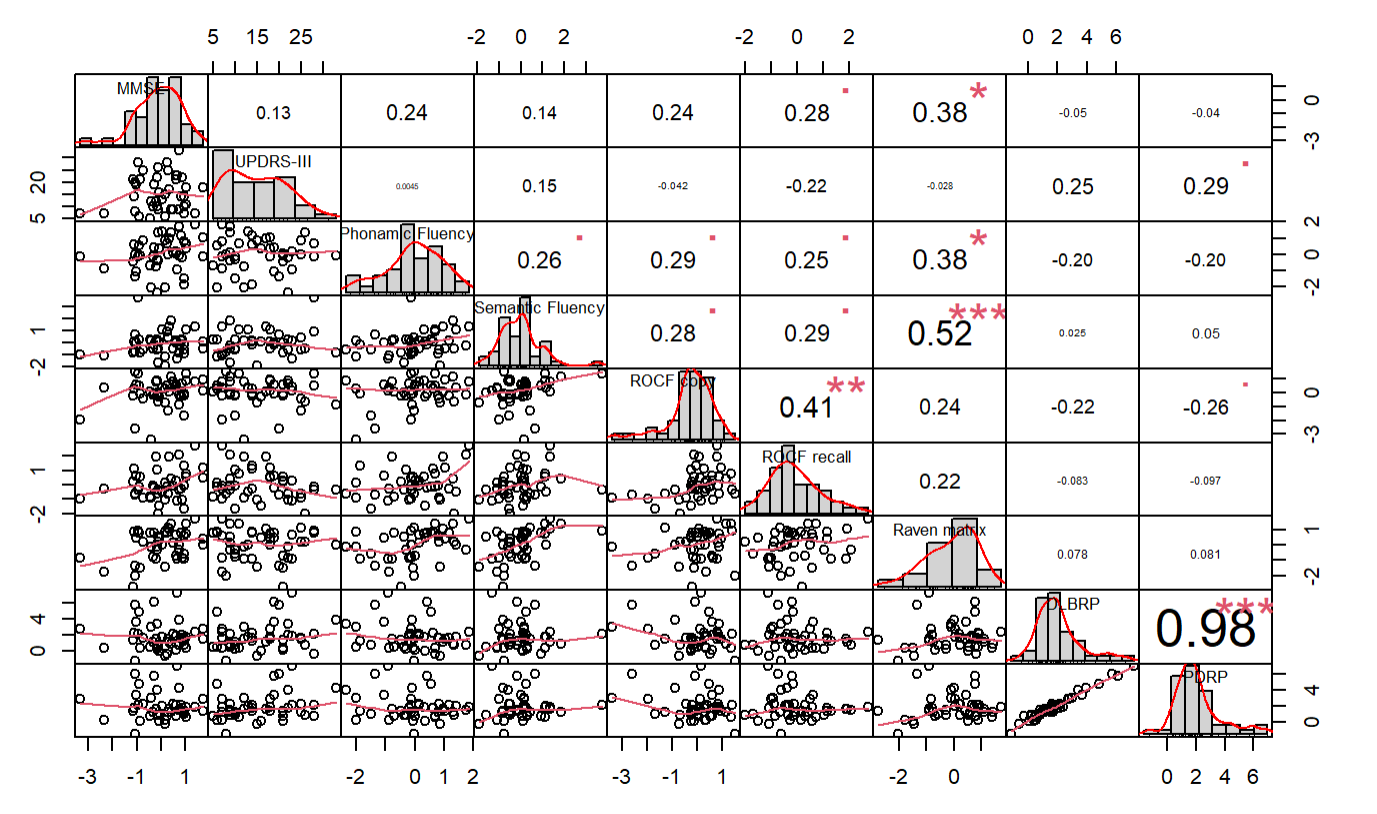


**Figure S2. Correlation between clinical variables and PDRP and DLBRP z-scores in PD.**


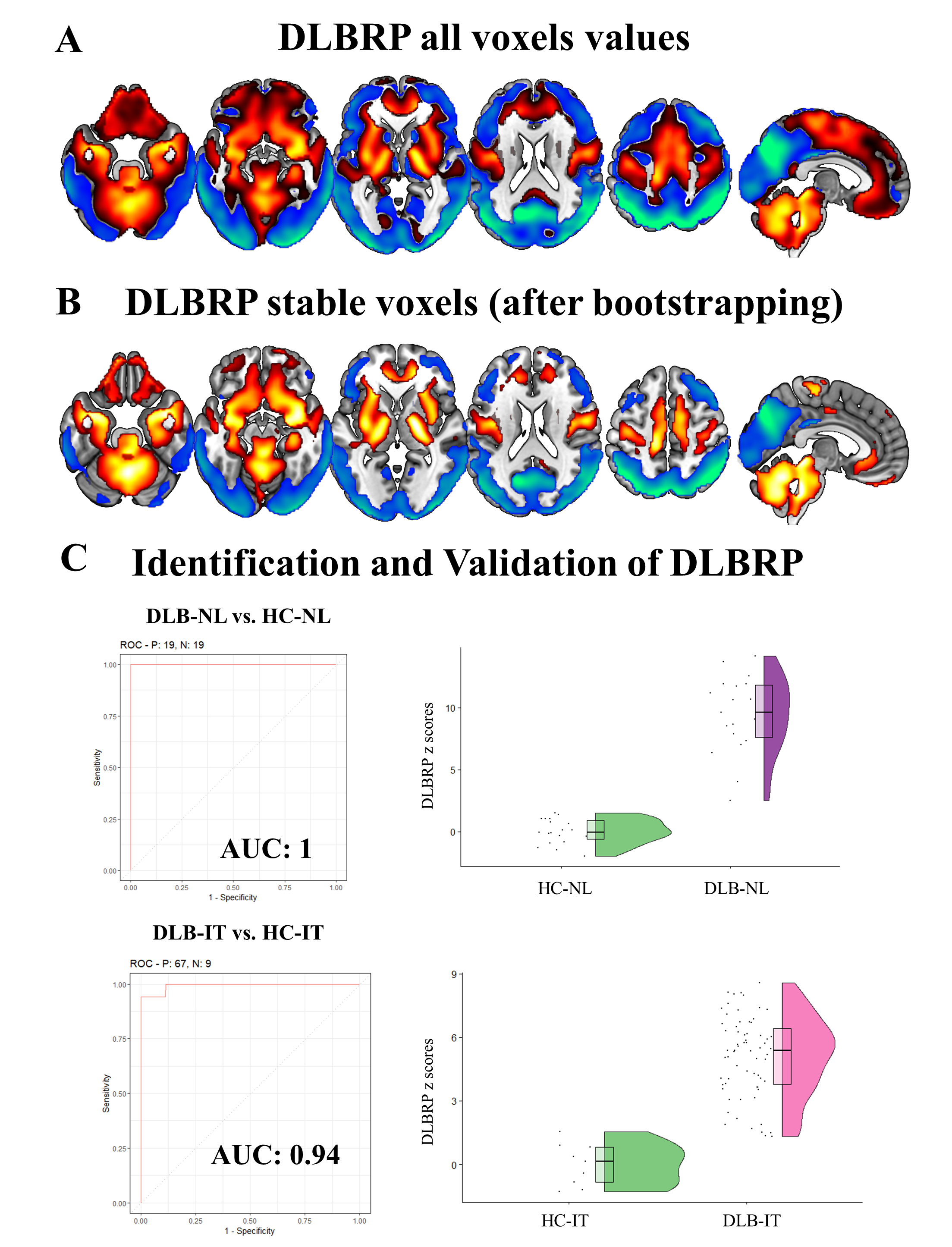


**Figure S3** **DLBRP topography and validation in the Italian cohort.** The DLBRP derived from the Netherlands cohort of patients (N=19 HC and N=19 DLB) is shown at the top of the figure. All voxel (A) and stable values (B) are overlaid on a T1 MR template. The discrimination performance and z-scores expressions in identification (NL) and validation (IT) cohorts are represented throughout ROCs curves and raincloud graphs, respectively (C). Abbreviations: DLBRP: Dementia with Lewy Bodies -related pattern; HC: healthy controls; AUC: Area Under the Curve; vs: versus.


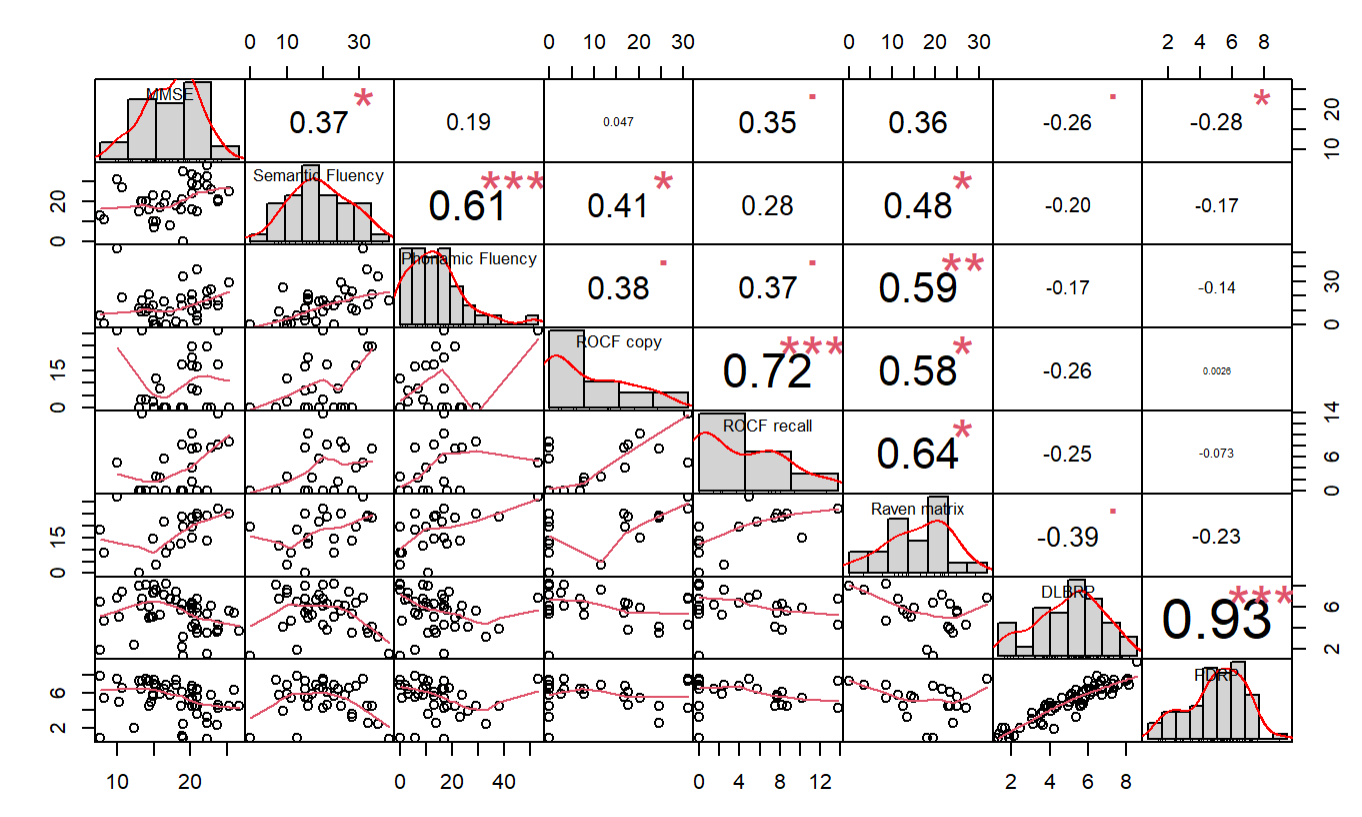


**Figure S4. Correlation between clinical variables and PDRP and DLBRP z-scores in DLB.**


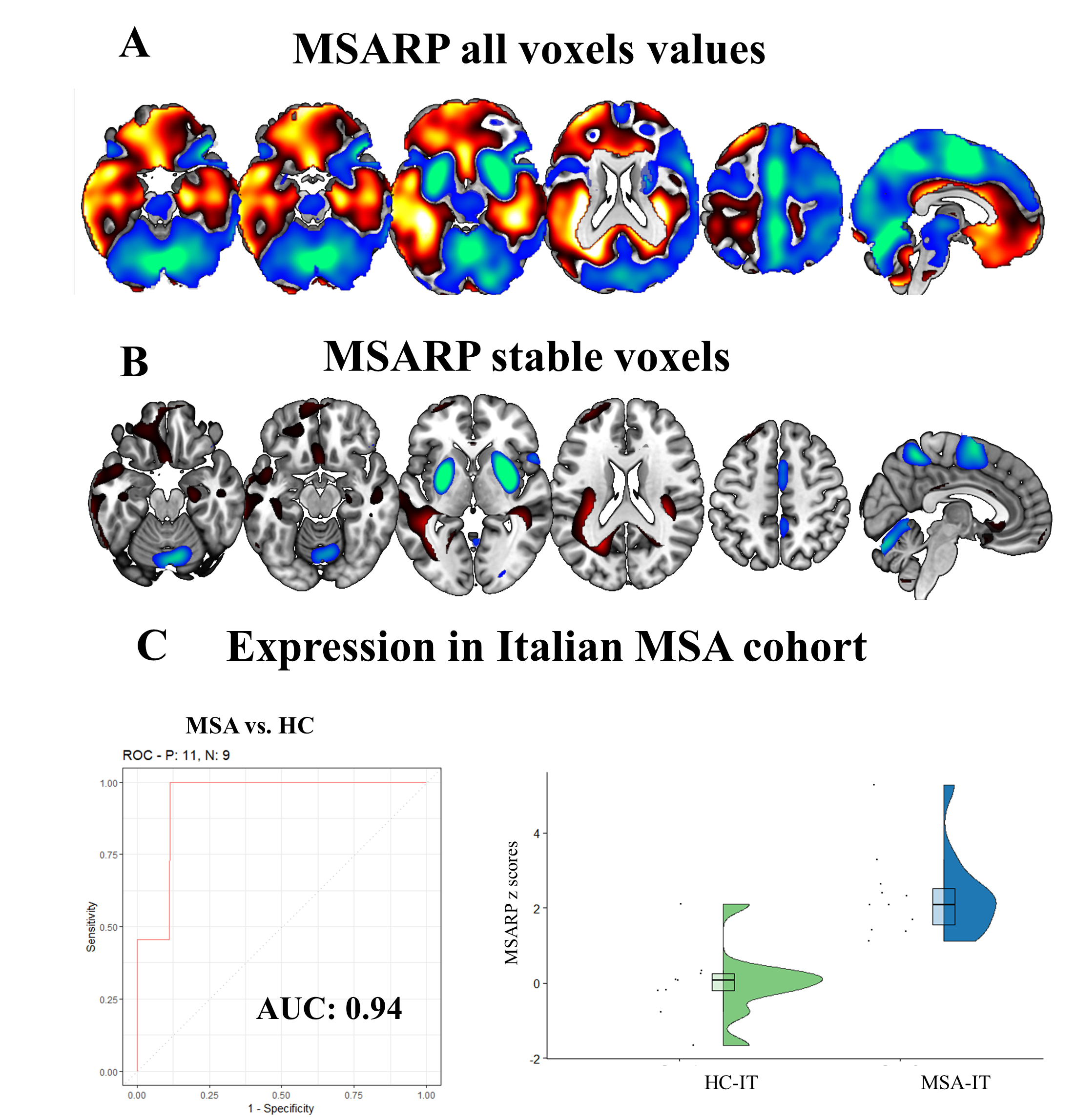


**Figure S5** **MSARP topography and validation in the Italian cohort.** All voxel (A) and stable values (B) are overlaid on a T1 MR template. The discrimination performance and z-scores expressions in the validation (IT) cohort is represented throughout ROCs curves and raincloud graphs, respectively (C). Abbreviations: MSARP: Multiple System Atrophy-related pattern; HC: healthy controls. AUC: Area Under the Curve; vs: versus.


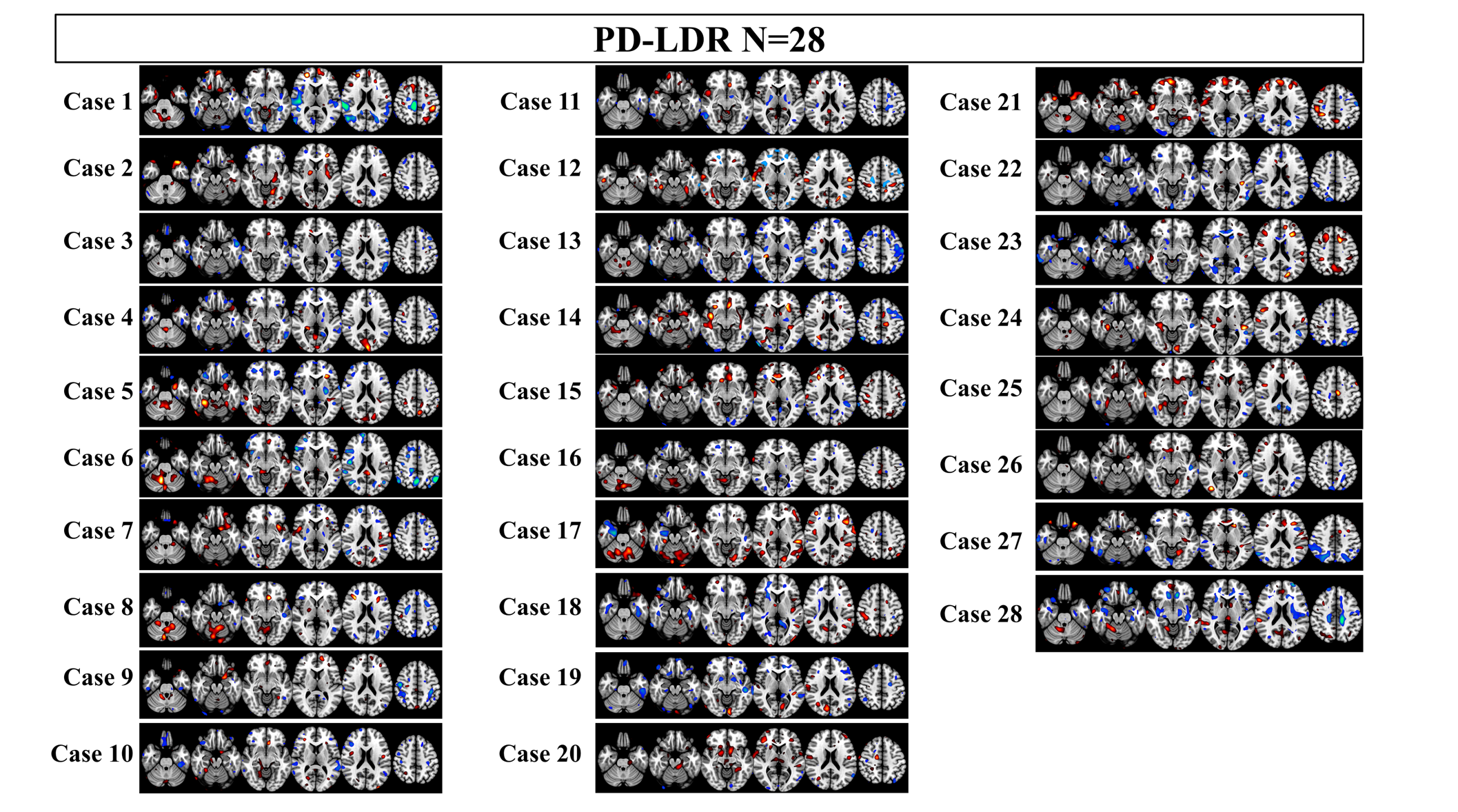


**Figure S6 Single-subject maps of hypo and hypermetabolism in PD patients cognitively stable ove time.**

**
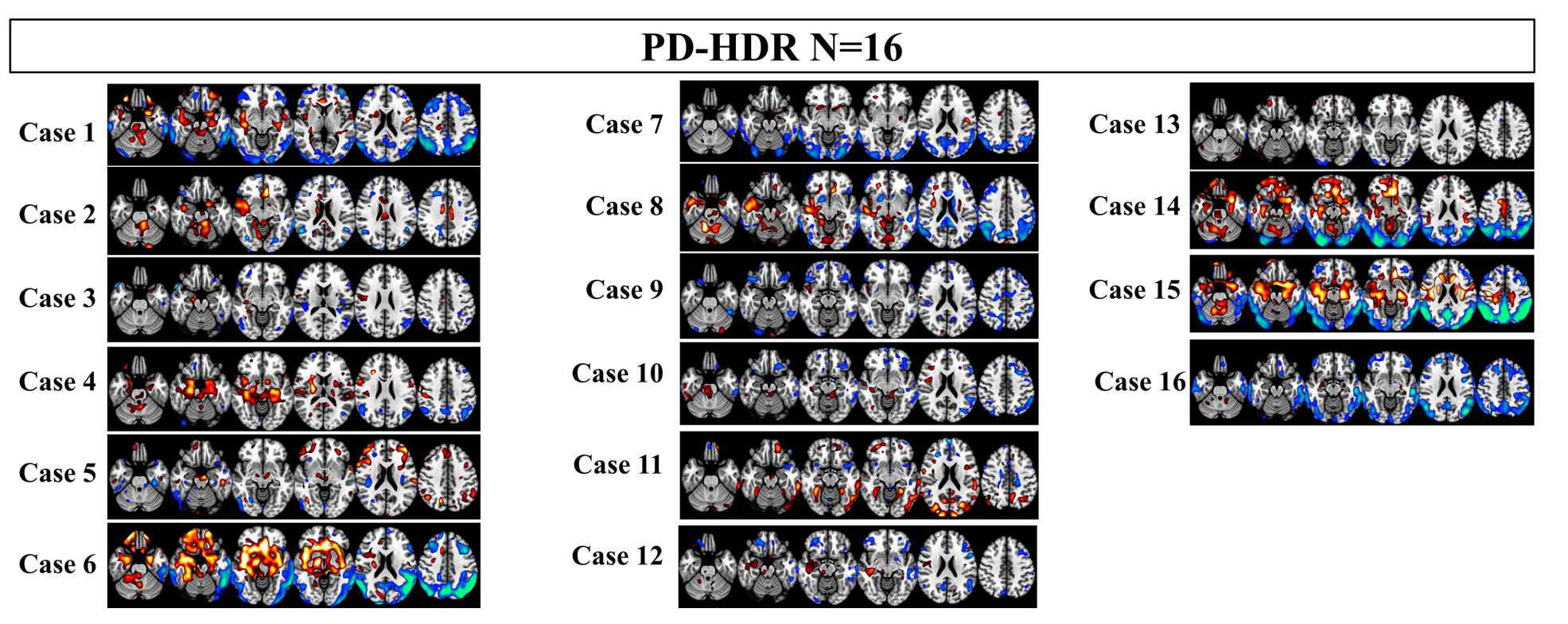
**

**Figure S7 Single-subject maps of hypo- and hypermetabolism in PD-HDR patients.**

**References**

1. Della Rosa PA, Cerami C, Gallivanone F, Prestia A, Caroli A, Castiglioni I, et al. A Standardized [18F]-FDG-PET Template for Spatial Normalization in Statistical Parametric Mapping of Dementia. Neuroinformatics. Springer; 2014;12:575–93.

2. Gallivanone F, Della PAR, Perani D, Gilardi MC, Castiglioni I. The impact of different 18FDG PET healthy subject scans for comparison with single patient in SPM analysis. Q J Nucl Med Mol imaging Off Publ Ital Assoc Nucl Med (AIMN)[and] Int Assoc Radiopharmacol (IAR),[and] Sect Soc of. 2017;61:115–32.

3. Perani D, Della Rosa PA, Cerami C, Gallivanone F, Fallanca F, Vanoli GE, et al. Validation of an optimized SPM procedure for FDG-PET in dementia diagnosis in a clinical setting. NeuroImage Clin. Elsevier B.V.; 2014;6:445–54.

4. Della Rosa PA. A Standardized [18F]-FDG-PET Template for Spatial Normalization in Statistical Parametric Mapping of Dementia. Neuroinformatics. Humana Press Inc; 2014;12:575–93.

5. Presotto L, Ballarini T, Caminiti SP, Bettinardi V, Gianolli L, Perani D. Validation of 18 F–FDG-PET Single-Subject Optimized SPM Procedure with Different PET Scanners. Neuroinformatics. Springer; 2017;15:151–63.

6. Caminiti SP, Sala A, Presotto L, Chincarini A, Sestini S, Perani D, et al. Validation of FDG-PET datasets of normal controls for the extraction of SPM-based brain metabolism maps. Eur J Nucl Med Mol Imaging. Springer; 2021;1–14.

7. Caminiti SP, Sala A, Iaccarino L, Beretta L, Gianolli L, Iannaccone S, et al. Brain glucose metabolism and connectivity support current diagnostic criteria for Lewy Body Dementia. Eur J Neurol. WILEY 111 RIVER ST, HOBOKEN 07030-5774, NJ USA; 2018. p. 346.

8. Pilotto A, Premi E, Caminiti SP, Presotto L, Turrone R, Alberici A, et al. Single-subject SPM FDG-PET patterns predict risk of dementia progression in Parkinson disease. Neurology. 2018;90:e1029–37.

9. Caminiti SP, Alongi P, Majno L, Volontè MA, Cerami C, Gianolli L, et al. Evaluation of an optimized [18F] fluoro‐deoxy‐glucose positron emission tomography voxel‐wise method to early support differential diagnosis in atypical Parkinsonian disorders. Eur J Neurol. Wiley Online Library; 2017;24:687-e26.

10. Sala A, Caprioglio C, Santangelo R, Vanoli, Giovanna Emilia Iannaccone S, Magnani G, Perani D. Brain metabolic signatures across the Alzheimer’s disease spectrum. Eur J Nucl Med Mol Imaging. Elsevier; 2019;In press.

11. Cerami C, Dodich A, Greco L, Iannaccone S, Magnani G, Marcone A, et al. The role of single-subject brain metabolic patterns in the early differential diagnosis of primary progressive aphasias and in prediction of progression to dementia. J Alzheimer’s Dis. IOS Press; 2017;55:183–97.

12. McKeith IG, Boeve BF, Dickson DW, Halliday G, Taylor J-P, Weintraub D, et al. Diagnosis and management of dementia with Lewy bodies: Fourth consensus report of the DLB Consortium. Neurology. AAN Enterprises; 2017;89:88–100.

13. Pagonabarraga J, Kulisevsky J, Llebaria G, Garcia-Sanchez C, Pascual-Sedano B, Martinez-Corral M, et al. PD-HDR-Short Screen: a brief cognitive test for screening dementia in Parkinson’s disease. Mov Disord. Movement Disorders Unit, Neurology Department, Sant Pau Hospital, Autonomous University of Barcelona, Barcelona, Spain.; 2010;25:440–6.

14. Verbaan D, Jeukens-Visser M, Van Laar T, van Rooden SM, Van Zwet EW, Marinus J, et al. SCOPA-cognition cutoff value for detection of Parkinson’s disease dementia. Mov Disord. Department of Neurology, Leiden University Medical Center, Leiden, The Netherlands. D.Verbaan@lumc.nl: Movement Disorder Society; 2011;26:1881–6.

15. Spetsieris P, Ma Y, Peng S, Ko JH, Dhawan V, Tang CC, et al. Identification of disease-related spatial covariance patterns using neuroimaging data. JoVE (Journal Vis Exp. 2013;e50319.

16. Akaike H. A new look at the statistical model identification. IEEE Trans Automat Contr. Ieee; 1974;19:716–23.

17. Meles SK, Kok JG, Renken RJ, Leenders KL. From Positron to Pattern: A Conceptual and Practical Overview of 18F-FDG PET Imaging and Spatial Covariance Analysis. PET SPECT Neurol. Springer; 2021. p. 73–104.
